# Personalized structural image analysis in patients with temporal lobe epilepsy

**DOI:** 10.1038/s41598-017-10707-1

**Published:** 2017-09-07

**Authors:** Christian Rummel, Nedelina Slavova, Andrea Seiler, Eugenio Abela, Martinus Hauf, Yuliya Burren, Christian Weisstanner, Serge Vulliemoz, Margitta Seeck, Kaspar Schindler, Roland Wiest

**Affiliations:** 1 Support Center for Advanced Neuroimaging (SCAN), University Institute for Diagnostic and Interventional Neuroradiology, Inselspital Bern, University of Bern, Bern, Switzerland; 2 Sleep-Wake- Epilepsy-Center, Department of Neurology, Inselspital Bern, University of Bern, Bern, Switzerland; 3 Epilepsy Clinic, Tschugg, Switzerland; 4 University Hospital of Psychiatry, University of Bern, Bern, Switzerland; 5 Presurgical Epilepsy Evaluation Unit, Neurology Department, University Hospital of Geneva, Geneva, Switzerland

## Abstract

Volumetric and morphometric studies have demonstrated structural abnormalities related to chronic epilepsies on a cohort- and population-based level. On a single-patient level, specific patterns of atrophy or cortical reorganization may be widespread and heterogeneous but represent potential targets for further personalized image analysis and surgical therapy. The goal of this study was to compare morphometric data analysis in 37 patients with temporal lobe epilepsies with expert-based image analysis, pre-informed by seizure semiology and ictal scalp EEG. Automated image analysis identified abnormalities exceeding expert-determined structural epileptogenic lesions in 86% of datasets. If EEG lateralization and expert MRI readings were congruent, automated analysis detected abnormalities consistent on a lobar and hemispheric level in 82% of datasets. However, in 25% of patients EEG lateralization and expert readings were inconsistent. Automated analysis localized to the site of resection in 60% of datasets in patients who underwent successful epilepsy surgery. Morphometric abnormalities beyond the mesiotemporal structures contributed to subtype characterisation. We conclude that subject-specific morphometric information is in agreement with expert image analysis and scalp EEG in the majority of cases. However, automated image analysis may provide non-invasive additional information in cases with equivocal radiological and neurophysiological findings.

## Introduction

High-resolution T1-weighted magnetic resonance imaging (MRI) is part of todays epilepsy protocol recommendations^1^, since they allow readers to identify structural abnormalities associated with temporal lobe epilepsy (TLE). Beyond expert rating, these datasets can be further used for computer-assisted quantitative image analysis to generate rater-independent biological fingerprints. In contrast to cohort-based identification of aberrant large-scale network structures, these fingerprints may provide additional diagnostic information for the individual patient suffering from epilepsy^2,3^ if expert readings and electroencephalographic (EEG) findings are equivocal.

Large-scale structural abnormalities suggest a common structural framework for TLE^4–6^. Several neuroimaging studies implicate that these abnormalities are not restricted to the hippocampus ipsilateral to seizure onset, but extend along the cingulate cortex, insula, thalamus and frontal lobes, even on the contralateral hemisphere^7–17^. While these findings pose a potential role for image-based classification, widespread application on the individual level is hampered by large inter-subject and cohort-dependent variability^7^. Findings are biased by sex, age, duration and hemispheric lateralization of the epilepsy subtype or by combinations with other conditions, as e.g. neurodegenerative disease.

So far, studies on the potential impact of automated analysis on individual diagnosis have provided conflicting results. While some authors demonstrated an additional yield of voxel based morphometry (VBM) for analysis^2,18–24^, others stressed the poor sensitivity and specificity of morphometric grey matter (GM) alterations^7,25^. Despite these limitations, Thesen *et al*.^26^ and Hong *et al*.^3^ suggested surface based morphometry (SBM) to detect focal cortical dysplasias in candidates for epilepsy surgery. Studies that demonstrated the feasibility of morphometric features (e.g. GM volume, cortical thickness, surface areas or curvatures) to classify patients with psychiatric disorders and epilepsy have recently been reported^27–31^.

In the present study, we suggest an automated analysis pipeline and statistical framework^32^ to generate personalised reports of brain morphometry without user intervention using the free software packages FSL^33^ and FreeSurfer^34,35^. The localizing value of the morphometric reports were compared against those of expert ratings of scalp EEG and MRI. Regional morphometric parameters of cortical and subcortical structures and their interhemispheric asymmetries (N = 2,976 features altogether) were extracted from single patient scans and statistically compared to 323 healthy control datasets accounting for age, sex and scanning parameters. The feasibility and validity of the proposed framework were evaluated on 47 T1-weighted high-resolution images from 37 patients with mesial temporal lobe epilepsies and hippocampal sclerosis (MTLE-HS) or lateral temporal lobe epilepsies (LTLE). FDG-PET or intracranial EEG information was not part of the standard evaluation programm at our center. Our study assessed the following questions: (1) How frequently are structural abnormalities obscured to visual rating? (2) Do structural abnormalities contribute to lateralization and sublobar determination of the seizure onset zone or of the resection site in individual patients?

## Results

### Clinical data and quality control

Clinical presentation and MR examinations of patients are summarized in Table S1 of the Supporting Information (SI). Statistical differences between subtypes of temporal lobe epilepsies were limited to the age distribution. Patients in the right LTLE subtype were significantly younger than in both MTLE-HS subtypes (Kruskal-Wallis test and post-hoc pair-wise Mann-Whitney-Wilcoxon tests). 5/47 MRIs (11%) did not pass our quality assessment due to failure of reconstruction or parcellation of the cortical surface and were excluded from further analysis, see Table S2 of the SI. Overall, 13/37 patients underwent epilepsy surgery (18/42 datasets with morphometry, i.e. 43%).

### Personalized analysis

**Figure 1:**
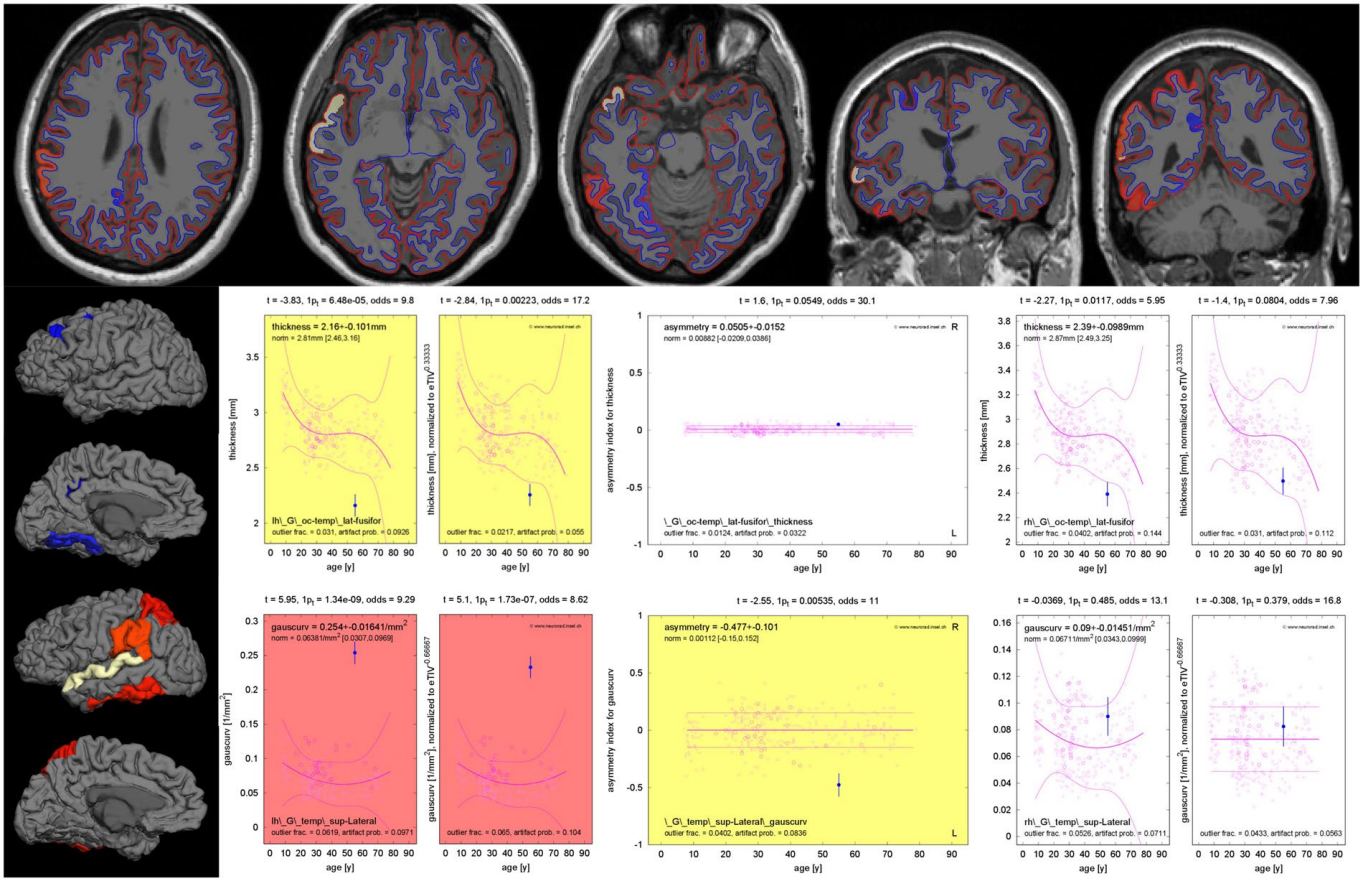
T1-weighted MRI and standardized result presentation for dataset P005, a MTLE-HS patient with left-sided seizure onset. In the top row three axial and two coronal slices are shown. The images are displayed according to “neurological” convention, i.e. the left side of the images corresponds to the left hemisphere. The gray-white matter interface found by FreeSurfer is displayed as a blue and the pial surface as a red line. Voxels corresponding to surface parcellations with abnormally high Gaussian curvature are highlighted with red-to-yellow coloring and voxels corresponding to reduced cortical thickness with blue-to-white coloring. The mean cortical thickness (middle row) was reduced in the left lateral occipito-temporal gyrus with and without eTIV normalization (p = 0.002). With odds of 17.2 the confidence in a valid (vs. artefactual) observation was high, especially after eTIV normalization. The corresponding structure on the right hemisphere and the asymmetry index were still within the normal range. The Gaussian curvature (bottom row) of the lateral aspect of the superior temporal gyrus was asymmetric (p = 0.005), with highly significantly increased curvature on the left (p < 10^−6^). Again, odds >8 gave confidence in reliable observations.

For illustration, a case of left-sided MTLE-HS is provided in Fig. 1 (dataset P005). Scalp EEG findings were not lateralizing. Expert analysis of the MRI identified left-hemispheric hippocampal as well as cortical atrophy (elevated cerebro-spinal fluid (CSF) and reduced GM volumes, pronounced in the left parietal and occipital lobe). The right hippocampus was considered normal by expert analyis. Automated morphometric analysis revealed reduced hippocampal volume on the left (2.1 ± 0.2 ml, volume estimate with uncertainty derived from repeated MRIs in the healthy control database^32^, p = 0.001). The volume of the left amygdala was at the lower end of the normal range (0.9 ± 0.2 ml, p = 0.034). The corresponding volumes on the right side were normal (hippocampus: 3.8 ± 0.3 ml, amygdala 1.5 ± 0.2 ml). Asymmetry of the hippocampus (p < 10^−14^), the pallidum (p < 10^−16^) and the putamen (p < 10^−8^, each of them smaller in the left hemisphere) were most significant. Morphometric analysis of cortical surface parcellations revealed widespread unilateral changes on the left hemisphere.

### Elevated abnormality rates in patients with temporal lobe epilepsy

We detected significantly higher abnormality rates in patients with TLE than in a leave-one-out cross-validation dataset of healthy controls^32^ (Table 1). Moreover, in datasets that had to be excluded due to quality issues, the “abnormality detection rate” was erroneously three times higher than in the datasets passing the checks. A more detailed analysis of the different morphometric parameters (not shown) revealed that asymmetry of hippocampal volumes was the most frequent observation in MRIs of individual patients with TLE (detected in 48% of datasets after normalization to the estimated total intracranial volume, eTIV), followed by volume loss of the left pallidum (21%) and ipsilateral hippocampus (17%). The asymmetry indices of volume segmentations (15% overall) were about six times more frequently abnormal in patients with TLE than in healthy subjects. Hippocampal asymmetry was significant in 83% of datasets with MTLE-HS but in none with LTLE. This difference between TLE subtypes was highly significant (p < 10^−5^, randomization test).

**Table 1:** Total count and percentage of regional uncorrected (p < 0.01) and FDR corrected morphometric abnormalities in MRIs of TLE patients.

		used datasets: 42 MRIs from 32 TLE patients			excluded datasets: 5 MRIs from 5 TLE patients		
		test count	p_uncorr_ < 0.01	p_FDR_ < 0.01	test count	p_uncorr_ < 0.01	p_FDR_ < 0.01
raw	count	83454	4041	1514	9935	1197	610
	percentage		4.84%	1.81%		12.05%	6.14%
	p (healthy controls)		**0**	**0**		**0**	**0**
	p (QC passed)		n.a.	n.a.		**0**	**0**
normalized	count	83454	4109	1553	9935	1300	622
	percentage		4.92%	1.86%		13.09%	6.26%
	p (healthy controls)		**0**	**0**		**0**	**0**
	p (QC passed)		n.a.	n.a.		**0**	**0**
asymmetry	count	41412	1456	326	4930	544	180
	percentage		3.52%	0.79%		11.03%	3.65%
	p (healthy controls)		**0**	**0**		**0**	**0**
	p (QC passed)		n.a.	n.a.		**0**	**0**

A binomial model was used to compare the empirical detection rates to the expectations in healthy controls^32^. In patients they were highly significantly larger than in a leave-one-out cross-validation in healthy controls with p-values zero to machine precision. For datasets that did not pass the quality checks we also compared to the detection rate in high quality datasets of TLE patients. In excluded datasets the detection rates were highly significantly elevated.

Abbreviations: FDR, false discovery rate; n.a., not applicable; QC, quality check; uncorr, uncorrected.

### Relation with epilepsy surgery

In patients who underwent successful epilepsy surgery, structural abnormalities were present at the site of resection on a sublobar level in 10/14 datasets that passed quality control (71%, see Table 2). All these detections were considered specific, since at least one of the three most significant abnormalities overlapped with the resected epiletogenic zone). There was a strong trend towards higher agreement in MTLE-HS than in LTLE (p = 0.015, randomization test). In surgery cases with consistent expert MRI and EEG rating an overlap of morphometric detections with the resection site was found in 5/5 datasets (100%). The overlap fraction in equivocal datasets (3/5 = 60%) was not significantly smaller (p = 0.333, exact test).

**Table 2:** Calculation of sensitivities and specificities from expert MRI and EEG analysis, see Table S2 of the SI for details.

MRI dataset	patient	morphometry: sensitivity			morphometry: specificity			agreement
		vs. expert MRI	vs. surgery	vs. EEG	vs. expert MRI	vs. surgery	vs. EEG	expert MRI vs. EEG
MTLE-HS left								
P001	1	1	–	1	1	–	1	1
P002	1	1	–	1	1	–	0	
P003	2	1	1	0	1	1	0	0
P004	2	1	1	0	1	1	0	
P005	2	1	1	0	1	1	0	
P006	3	1	–	1	1	–	1	1
P007	4	1	1	1	1	1	1	1
P008	5	1	–	1	1	–	1	1
P009	5	0	–	0	0	–	0	
P010	6	1	1	1	1	1	1	1
P011	6	1	1	1	1	1	1	
P012	7	0	–	0	0	–	0	1
P013	8	1	1	1	1	1	1	1
P014	9	1	–	–	1	–	–	–
P015	10	1	–	1	1	–	1	1
P016	11	1	–	1	1	–	1	1
P017	12	1	–	1	1	–	1	1
P018	13	1	–	1	1	–	1	0
sensitivity/specificity		0.889	1.000	0.706	0.888	1.000	0.647	0.833
MTLE-HS right								
P019	14	–	–	–	–	–	–	1
P020	15	–	–	–	–	–	–	1
P021	16	–	–	–	–	–	–	1
P022	17	1	1	1	1	1	1	1
P023	18	1	–	1	1	–	1	1
P024	19	–	–	–	–	–	–	1
P025	20	0	–	–	0	–	–	–
P026	21	1	–	–	1	–	–	–
P027	22	1	–	0	0	–	0	0
P028	23	1	–	–	1	–	–	–
sensitivity/specificity		0.833	1.000	0.667	0.667	1.000	0.667	0.857
LTLE left								
P029	24	–	–	0	–	–	0	–
P030	25	–	0	0	–	0	0	–
P031	25	–	0	0	–	0	0	
P032	26	–	–	–	–	–	–	–
P033	27	1	–	1	0	–	1	0
P034	27	1	–	1	0	–	0	
P035	27	0	–	0	0	–	0	
P036	28	0	–	0	0	–	0	1
P037	29	1	–	–	1	–	–	–
P038	30	–	–	1	–	–	1	–
sensitivity/specificity		0.600	0.000	0.375	0.200	0.000	0.250	0.500
LTLE right								
P039	31	1	–	0	0	–	0	0
P040	32	0	0	0	0	0	0	0
P041	32	1	0	1	1	0	1	
P042	33	1	1	–	1	1	–	–
P043	33	1	1	–	1	1	–	
P044	34	–	–	0	–	–	0	–
P045	35	0	–	–	0	–	–	–
P046	36	–	–	0	–	–	0	–
P047	37	1	–	1	1	–	1	1
sensitivity/specificity		0.714	0.500	0.333	0.571	0.500	0.333	0.333
overall sensitivity/specificity		0.806	0.714	0.559	0.694	0.714	0.500	0.750

Morphometric findings were counted as *sensitive* if at least one detection with L_r_ ≥ 2 (equation (2) in the Methods section) was consistent with a lesional finding by expert MRI reading, the surgical resection in case of favorable outcome (Engel class I and II) and follow-up for at least six months, or lateralizing signs in expert EEG reading. More strictly, morphometry was counted as *specific* if any of the three most prominent detections with L_r_ ≥ 2 was consistent. Overall sensitivities and specificities were calculated as the fraction of consistent datasets in all lesional MRIs, in all datasets with subsequent surgery or in all datasets with focal EEG, respectively.

Matches are indicated by “1” and disagreement by “0”. The dash indicates that information was not available or not applicable.

### Comparison with expert image analysis

As expert image reading is part of the routine clinical workup of epilepsy patients, we assessed the performance of our framework to detect morphometric alterations with expert detections as ground truth (GT), see SI for details. The essence of abnormalities detected by our quantitative analysis pipeline in comparison to expert reading of the MRI and scalp EEG is provided in Table 2 and further information is available in Table S2 of the SI. Expert raters identified 6/37 patients as non-lesional (7/47 of MRI datasets, i.e. 15%). In contrast, automated morphometric analysis was normal only in two MRI datasets (2/42 = 5%). The overall detection rate of structural abnormalities was not significantly different between automated analysis and readers (p = 0.082, randomization test). While the expert readers reported a higher fraction of non-lesional MRIs in LTLE than in MTLE-HS (p = 0.001), automated detection was independent from the TLE subtype.

The overall sensitivity of automated detections with L_r_ ≥ 2 according to equation (2) (see Methods section for details) to abnormalities reported by the expert readers was 0.806 (29/36 lesional MRIs that passed quality control, see Table 2). The overall specificity was 0.694 (25/36 MRIs). Specificity tended to be higher in MTLE-HS than in LTLE (p = 0.019, randomization test), while sensitivities were similar. Top 3 abnormalities with L_r_ ≥ 2 that were overlooked by the expert reader were detected in 36/42 MR datasets (86%).

**Figure 2:**
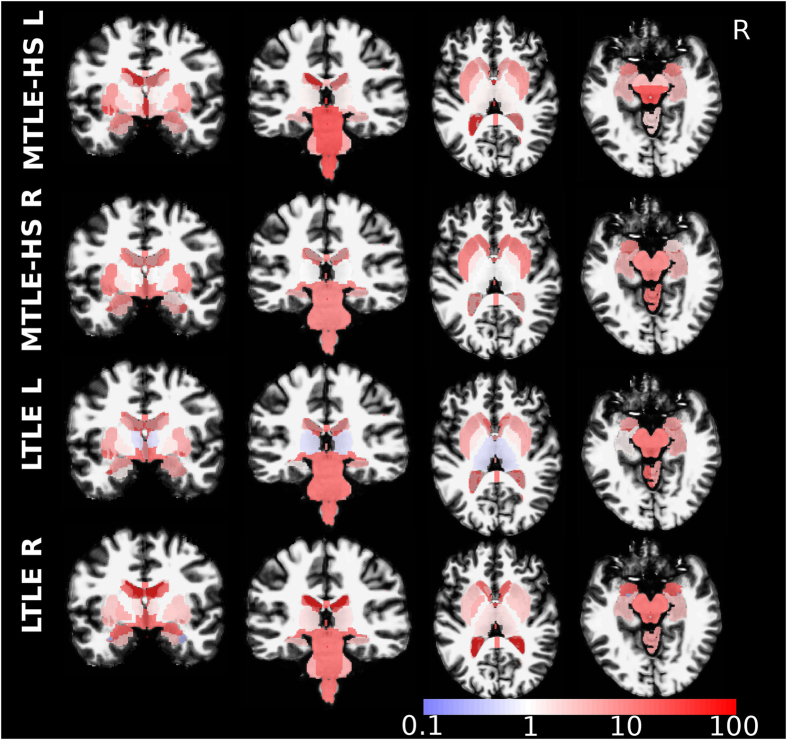
Diagnostic odds ratio of morphometric detections in volume segmentations with respect to expert MRI assessment. Coronal and axial slices are in neurological orientation, i.e. the left hemisphere appears on the left.

**Figure 3:**
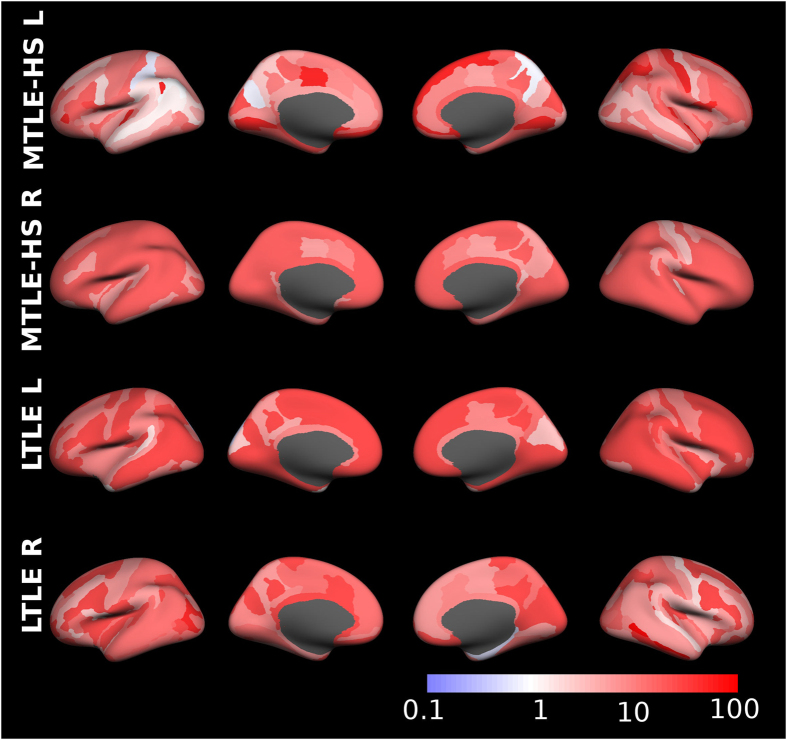
Diagnostic odds ratio of morphometric detections in surface parcellations with respect to expert MRI assessment. For better visibility of sulci the results are presented on inflated brain surfaces.

Regional diagnostic odds ratios (DOR, see Methods section for details) of automatic detections compared to the GT provided by expert reading are presented in Fig. 2 (volume segmentations) and Fig. 3 (surface parcellations). DOR ≫ 1 (red hue) indicate brain regions where the morphometric analysis matched the GT. The overall average DOR was 17.1 with range between 0.333 and 159. DORs ≤ 1 were observed in the thalamic nuclei and not specific for a TLE subtype. A subtype dependence of DOR was observed for surface parcellations, predominantly in the temporal and frontal lobes, see Fig. 3. In MTLE-HS with left-sided seizure onset the expert reported only a small fraction of abnormalities detected by the morphometry tool in the left lateral temporal lobe, which lowered the DOR.

Summaries of regional positive predictive values (PPV) are given in Figure S1 of the SI. In general, PPVs were moderate to small, (overall mean PPV was 0.113), meaning that the regional fraction of correct cases (“true positives” according to expert MRI assessment) among all cases rated abnormal by the morphometry tool (“test positives”) was considerably low. In contrast, negative predictive values (NPV) were in general very close to one, see Figure S2 of the SI, meaning that the vast majority of cases rated normal by the morphometry tool (“test negatives”) were also rated normal by the human expert (“true negatives”). The overall mean NPV was 0.974 and smallest values 0.6 < NPV < 0.7 were observed for atrophy of hippocampi and amygdalae as well as for volume increase of some ventricles, meaning that the informed expert did sometimes report abnormalities in these structures whereas the automated morphometry tool did not.

### MRI vs EEG lateralisation

Thirty out of 36 patients with available EEG information (corresponding to 39/46 of MRI datasets, i.e. 85%) presented with focal interictal abnormalities on routine scalp EEG (i.e. focal epileptiform signals and/or focal slowing), see Table S2 of the SI. The sensitivity and specificity of MR matches with the EEG lateralization were considerably low (0.559 and 0.500, see Table 2).

MRI and EEG localization by the expert overlapped in 18/24 patients (75%). This corresponded to 17 datasets with morphometry and in 14 cases automated detections were consistent with MRI and EEG (82%). However, in 25% of patients the expert ratings of the MRI were either non-lesional or EEG diffuse or both contradictory. In 9/11 of corresponding datasets with available morphometry (82%) detected abnormalities were sensitive for MR lesions according to the expert and in 4/11 datasets (36%) for EEG lateralization. In 4/11 cases some abnormalities were sensitive for MRI lesions and different ones for the EEG lateralization.

### Reproducibility of deviations from the norm

**Figure 4:**
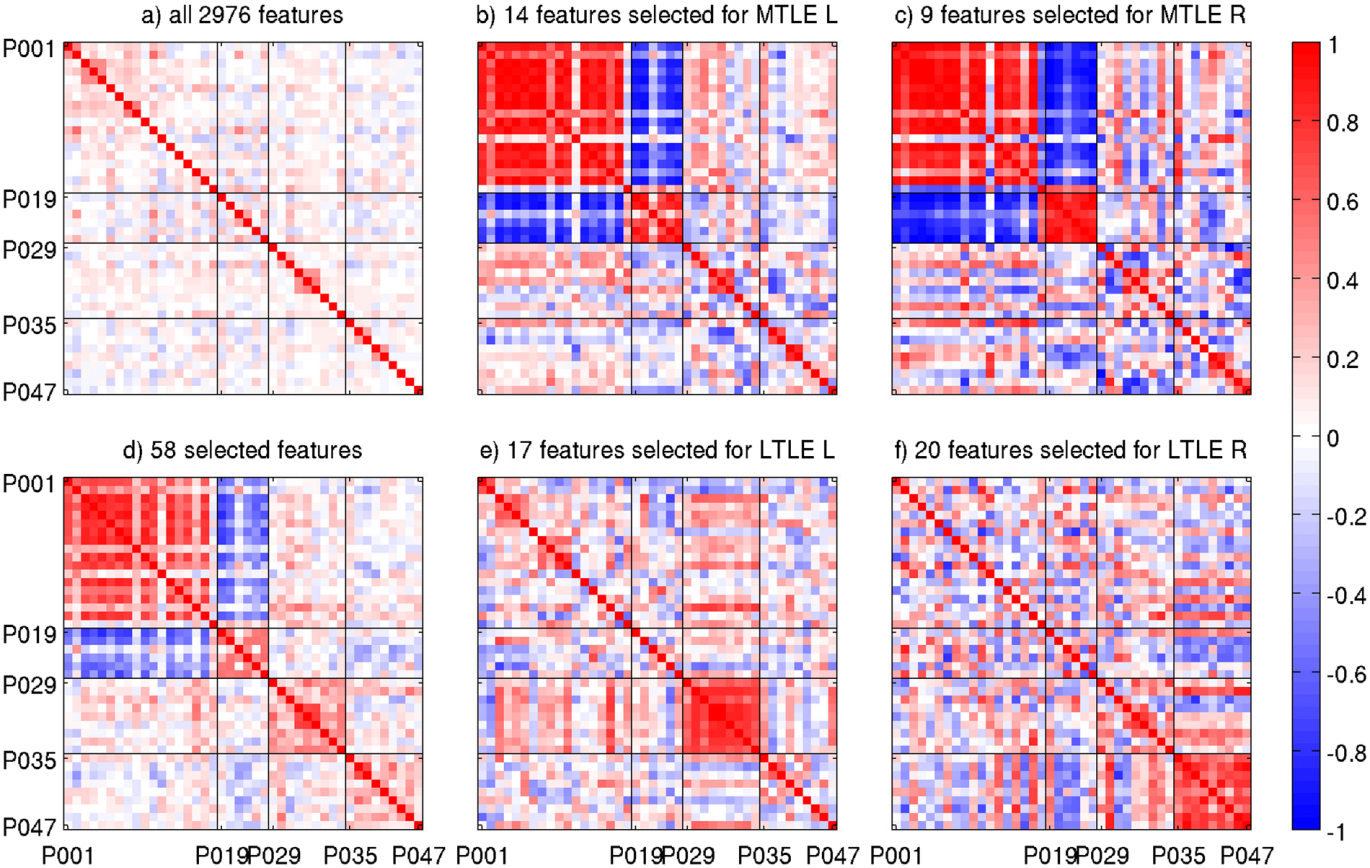
Pearson correlation matrices of feature vectors defined in equation (1). In panel a all 2,976 features are used, whereas panels b, c, e and f use only features representative for one TLE subtype. In panel d all features are used that were selected as representative for at least one subtype. Boundaries between datasets corresponding to different subtypes are indicated by black lines.

We constructed feature vectors of length 2,976 describing the direction and significance of regional deviations of morphometric parameters from the expectation in the healthy population (see Methods section for details) and assessed the reproducibility in repeated MRIs of the same patients by the Pearson correlation matrix (Fig. 4a). Within-patient correlation (median of the 12 off-diagonal matrix elements 0.436, 95% CI [−0.036,0.518]) was highly significantly larger than correlation between different patients (median of 849 matrix elements 0.032, CI [−0.183,0.273]) in a Mann-Whitney-Wilcoxon test (p < 10^−6^). Also, within-subtype correlation coefficients (repeated MRIs of the same patients excluded, median of 228 matrix elements 0.049, CI [−0.167,0.292]) were larger than between different TLE subtypes (median of 621 matrix elements 0.027, CI [−0.201,0.258], p < 10^−3^).

**Figure 5:**
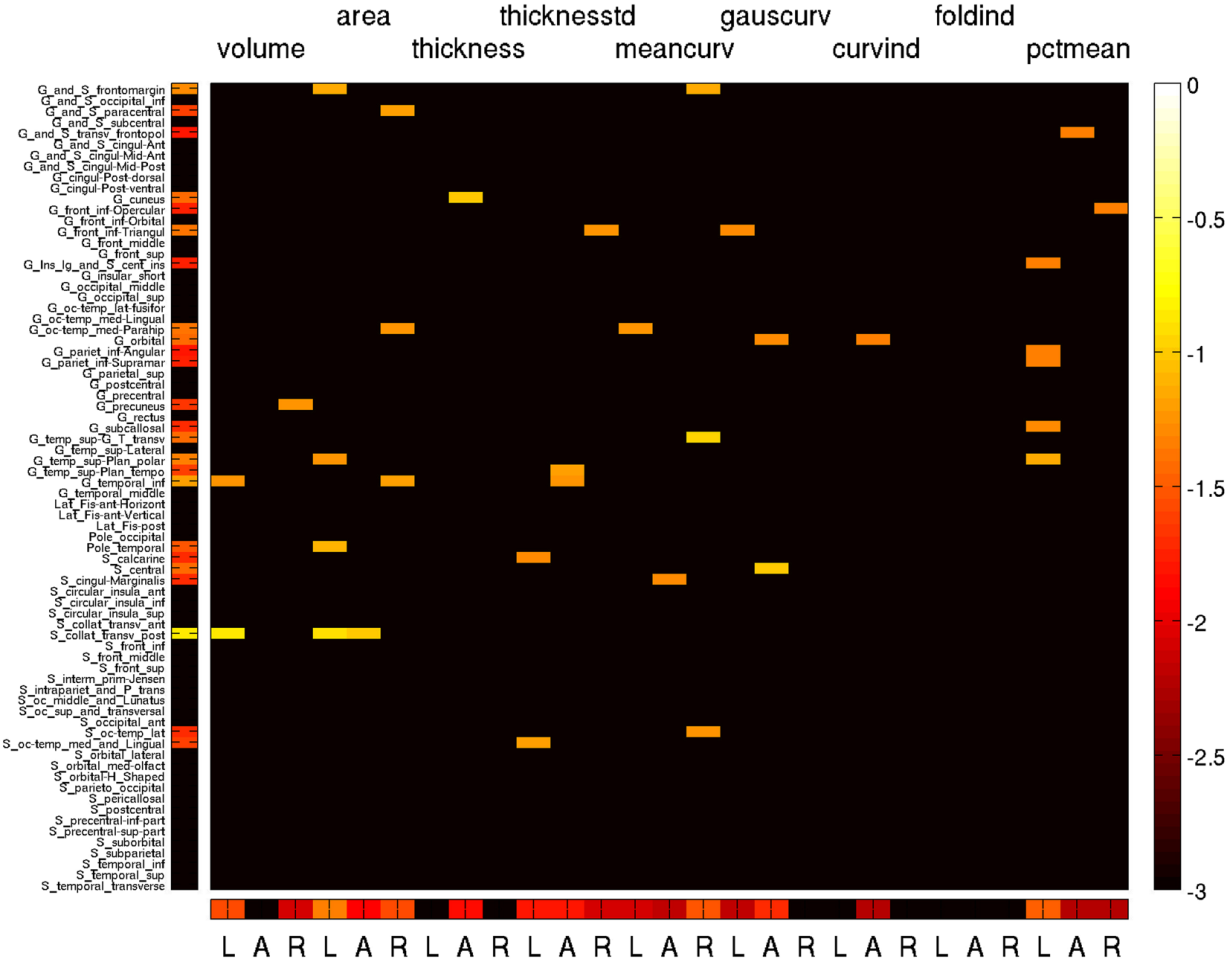
Cortical features of the Destrieux atlas selected to represent at least one of the TLE subtypes. The color coding represents the decadic logarithm of relative feature importances (values for all features add up to one). Abbreviations: A, asymmetry index; L/R, left/right; thicknessstd, standard deviation of cortical thickness; meancurv, mean curvature; gausscurv, Gaussian curvature; curvind, curvature index; foldind, folding index; pctmean, mean percentage change of the grey-white contrast.

After determining subsets of relevant features (see Methods section), block patterns became apparent in the correlation matrices (Fig. 4b,c,e,f). Two key features for subtype characterization were hippocampal asymmetry and left-hemispheric volume. Surface parcellation features were selected from all morphometric parameters and distributed over large parts of the cortex, see Fig. 5 for the Destrieux altlas and Figure S3 of the SI for the Desikan-Killiany atlas. In addition to parcellations of the temporal lobes also central and parietal parcellations were selected, see Figures S4 to S6 of the SI for mappings of subtype-wise z-values of SBM parameters.

Overall, 58/2976 features (1.9%) represented at least one TLE subtype. In contrast to the full feature vectors, the within-subtype correlation coefficients (median 0.347, CI [−0.124,0.869]) turned out highly significantly larger than between subtypes (median 0.009, CI [−0.627,0.335], p < 10^−64^). The MTLE-HS cases with left and right-sided seizure onset appeared highly anti-correlated in this feature selection (Fig. 4d).

## Discussion

Lack of apparent structural epileptogenic lesions (in 15% of datasets in our study) and equivocal EEG findings (in 25% of patients in our study) are frequent observations in patients who are considered candidates for epilepsy surgery. Expert MRI analysis using higher field strength and dedicated epilepsy protocols has improved the detection rates of structural epileptogenic lesions considerably^1,36^. Modern MR imaging enables high-resolution image acquisition within reasonable acquisition times, enabling additional postprocessing of the structural datasets. Here, we aimed to investigate the additional yield of automated volumetric and morphometric image analysis, using high-resolution T1-weighted sequences^32^.

### Main findings

In cases with consistent human MRI and EEG localization, agreement of morphometric abnormalities on a lobar level was 82%. In non-lesional or inconsistent datasets the morphometric abnormalities co-lateralized with EEG in 36% of cases. Partial overlaps with MRI lesions were 82% and with the resection site 60%.

The overall sensitivity and specificity of sublobar concordance with expert MRI inspection were 81% and 69% and larger for MTLE-HS than for LTLE. Widespread statistical abnormalities, even remote from the EEG focus, including abnormalities in the contralateral hemisphere were detected in the majority of datasets (86%), further supporting the concept of epilepsy as a widespread network disorder^4,5^.

Diagnostic odds ratios of automatic analysis using expert MRI as ground truth were in general high (Figs 2 and 3). The large negative vs. moderate positive predictive values of quantitative morphometric analysis (Figures S1 and S2 of the SI) fulfill the criteria of a screening test^37,38^. Volume segmentations and surface parcellations that were not flagged as suspicious by automated morphometric analysis were very likely also to be considered normal by the expert rater. In contrast, a positive detection through the morphometry pipeline did not necessarily imply that the region was also identified by the expert rater. During daily clinical workflow, such subtle morphometric changes^31,39^ may be overlooked despite being potential target regions that merit a second look by the human expert.

The morphometric feature vectors L_rm_ defined in equation (1) were highly reproducible and allowed to identify patients with repeated MRIs by elevated Pearson correlation coefficients (Fig. 4a). A similar observation has already been made by Rummel *et al*.^32^ for multiple sclerosis patients and healthy controls. As an outlook to forthcoming studies, we demonstrated that heuristic selection of a small number (1.9%) of representative features greatly enhanced the contrast between TLE subtypes (Fig. 4d). Importantly, the heuristic feature selection used hippocampal asymmetry in MTLE-HS patients, but was not limited to ipsilateral mesiotemporal structures. Rather, widespread and bilateral cortical features were selected (Fig. 5), including surface areas and curvatures, which easily escape expert inspection. We interpret this observation as a strong indication that the extracted features may serve as an excellent starting point for syndrome classification using machine learning approaches^40^.

### Limitations

One major limitation of this study is that our pipeline missed a proportion of hippocampal scleroses identified by the expert (8/31, i.e. 26%, see Table S2 of the SI). Expert raters detected predominantly signal changes on T2-weighted and FLAIR images, whereas morphometric analysis by FSL and FreeSurfer was restricted to T1-weighted images. The shortcoming of our morphometric analysis is consistent with Pardoe *et al*.^41^, who observed that manual hippocampus segmentation in patients with TLE yielded lower hippocampal volumes and a larger size difference between affected and non-affected hemispheres than automated segmentation. A similar observation was recently made by Kim *et al*.^42^, who also found that hippocampal developmental abnormalities and atrophy had a negative impact on the segmentation performance of three state-of-the-art automated methods (including FreeSurfer). Despite these possible negative biases in the methodology, abnormal hippocampal asymmetry or ipsilateral atrophy were the most frequent deviations of the patients observed in temporal lobe epilepsies compared to controls in our study. To overcome the current limitations of morphometric analysis based on FSL and FreeSurfer, specialized tools^43,44^ may further improve automated analysis. For these approaches, higher volumetric accuracies were reported for hippocampi and amygdalae and detection of hippocampal sclerosis was 100% in nine patients^43^. Alternatively, combinations with machine learning methods taking into account different imaging modalities and feature maps are expected to overcome this limitation in the near future.

## Conclusion

We aimed to investigate the applicability of case–control analyses to identify structural abnormalities in patients with epilepsy using surface-based morphometry and a framework of a customized statistical analysis. A similar approach with VBM volumes has previously been followed by Huppertz *et al*.^2,18,19^. Our approach did not outperform expert assessment in terms of lesion detection and subsequent classification of the underlying TLE subtype. In particular, our automated morphometry pipeline shares typical features of screening tests and must not be clinically applied without caution. Rather, automated detections must be followed-up with reinspection by the human experts. Notwithstanding, the fact that our heuristic feature selection enhanced subtype separation (see Fig. 4b–f) is an indication that widespread and subtle alterations escaping the expert’s eye carry information that might be exploited in future attempts to classification. Research in this direction is ongoing.

The fully automated analysis is computationally extensive (approximately ten hours for FreeSurfer and FSL, and two hours for statistics and figure generation), but it does not require time-consuming user intervention. In our study, launching the analysis and performing quality control took approximately three and ten minutes, respectively. Whereas these steps could be done by a technician, reviewing and interpreting the standardized result display requires an expert. It took approximately half an hour in our study. In our view, this means that incorporating the automated pipeline into clinical routine should be feasible without straining available resources. As it passes quality assessment in 89% of cases and can help to exclude non-suspicious brain regions, this tool could potentially be used to support the expert in the identification of structural abnormalities associated with structural epileptogenic lesions. Further studies including a larger cohort of surgery patients with different types of epilepsy are mandatory.

## Materials and Methods

This study was approved by the Ethics Committee of the Kanton of Bern in accordance with the Declaration of Helsinki. All patients and healthy controls signed informed consent for usage of their data for research. Any clinical decision making was done prior to and completely independent from the present study. For our retrospective analysis, MRI datasets were evaluated quantitatively, and MRI and EEG exams were re-assessed by neuroradiologists and neurologists. Experts were blinded to morphometric results and MR and EEG analysis techniques are described in detail in the SI. For comparison with automated detections, the neuroradiologist annotated regions with visually detectable abnormalities using the Destrieux atlas^45^ of surface parcellations.

### Patients and healthy controls

We analyzed high-resolution T1-weighted datasets of 37 patients with temporal lobe epilepsies scanned between 2009 and 2015 (17 male and 20 female, age range 18–76 years, see Table S1 of the SI for detailed patient characteristics and Table S3 for scanners and sequence parameters). Epilepsy surgery was not an inclusion criterion. For patients who underwent repeated scanning during their clinical workup at our institution, *all* available high-resolution T1-weighted MRI datasets were analyzed, resulting in a total of 47 datasets. The repeated MR datasets were used to evaluate the intra-patient reproducibility of our approach.

The normative database is described in detail in Rummel *et al*.^32^. It was generated from region-specific morphometric and volumetric parameters evaluated in a control group encompassing 323 high-resolution T1-weighted MRI datasets of 267 neurologically healthy subjects (142 datasets from male and 181 from female subjects, age range 7–79 years). The MRIs of healthy controls (HC) had been acquired during previous studies performed at the Inselspital Bern. Repeated MRIs from the same HCs within less than three years were used to estimate the region and parameter specific measurement errors^32^. The normative database was fully anonymized after its generation. Only age, sex, MR manufacturer/model and acquisition sequence were retained as confounding variables in anonymized form.

### Automated image processing and quality control

All image processing was performed on a quad-core workstation under Ubuntu Linux, release 14.04 LTS using the free software packages FSL and FreeSurfer. For statistics and result display according to Rummel *et al*.^32^, self-written Octave scripts were used (CR). Decisions were made at a significance level α = 0.01 and values p < 0.05 were interpreted as marginally significant (trends).

The total volumes of CSF, GM and WM were estimated using FSL (http://fsl.fmrib.ox.ac.uk/fsl/fslwiki/, version 5.0^33^). As additional volume parameters the whole brain volume (i.e. GM + WM) and the intracranial volume (ICV = GM + WM + CSF) were used. For estimation of the volumes of GM, WM and CSF segmentations including the hippocampus and the amygdala, the thalamus, the basal ganglia, the ventricles, the corpus callosum and the cerebellum we used FreeSurfer (https://surfer.nmr.mgh.harvard.edu, version 5.3.0), for details see Fischl *et al*.^34,35^. For surface-based morphometry, the interfaces between WM and GM as well as between GM and CSF were estimated with FreeSurfer and the cortex was parcellated automatically^46^ according to the atlases by Desikan *et al*.^47^ and Destrieux *et al*.^45^ providing 34 and 74 parcellations per hemisphere, respectively.

Abnormality detection is highly sensitive to errors occurring during FreeSurfer’s automatic surface tesselation, segmentation and parcellation procedures. Any patient datasets that did not pass the automated quality checks described in detail in the SI, and conditional secondary visual controls, were excluded from further analysis. In the healthy controls (N = 323) we did not perform these labor-intensive procedures. Rather, we rejected region and parameter specific statistical outliers, and estimated artifact probabilities as well as odds for valid vs. artifactual measurements following procedures described in detail in Rummel *et al*.^32^.

### Statistical analysis and standardized result presentation

To account for the physiological age-dependence affecting most morphometric parameters^48–53^, low order polynomial age trends were fitted to the estimates of all 323 members of the normative dataset according to Rummel *et al*.^32^. We calculated the probability *p* that a fit residue of observed magnitude occurred by chance, given the width of the distribution in healthy controls and the estimated measurement error.

Inter-individual differences in general head/brain size^54^ were accounted for by normalizing raw estimates of the regional morphometric parameters GM volume, cortical surface area, mean and standard deviation of the cortical thickness and all four curvature parameters to FreeSurfer’s estimated total intracranial volume (eTIV). We performed isometric normalization with different scaling exponents *n* to account for the different scaling relationships between morphometric parameters and head size (*n* = 1 for volumes, *n* = 2/3 for areas, *n* = 1/3 for thicknesses and accordingly for the curvature measures, see Rummel *et al*.^32^ for details). Grey-white contrast mainly depends on the MR sequence and thus it was instead regionally normalized to the same mean for each scanner-sequence combination. In brain regions appearing in both hemispheres, we also calculated asymmetry indices for all morphometric parameters. Their range is between −1 for extremely left-dominated and + 1 for extremely right-dominated structures, respectively. For regions with complete morphological symmetry, asymmetry indices equal zero.

To ease clinical translation of high-dimensional morphometric output, we generated standardized morphometry reports^32^. All regional morphometric parameters were compiled and displayed as a function of age with standardized layout (see Figure S7 of the SI). Brain regions and morphometric parameters with potential abnormalities were highlighted with a yellow figure background if deviations between the patient and the healthy controls had uncorrected significance p < 0.01. Correction for multiple comparisons during extensive testing (2,976 tests for raw and eTIV normalized parameters in each dataset) was performed using the false-discovery rate (FDR)^55^. Differences that remained significant after FDR correction were highlighted with a red figure background.

### Construction of feature vectors

To assess the agreement between automated morphometry and human rating, as well as between patients with repeated MRIs, we converted the normalized p-values of all brain regions *r* and morphometric parameters *m* into feature vectors:

1 $${{\rm{L}}}_{{\rm{rm}}}=\pm {\mathrm{log}}_{10}({{\rm{p}}}_{{\rm{rm}}})$$

of length 2,976 (60 whole brain partial volumes and volume segmentations, 34 plus 74 surface parcellations of the Desikan-Killiany and Destrieux atlases, evaluated on the left and right brain hemisphere plus the asymmetry index, all evaluated for 9 cortical morphometric parameters). In equation (1) the negative sign (resulting in positive *L* because p < 1) was used if the fit residue was positive (i.e. observation larger than expected), and the positive sign (negative *L*) if the residues were negative (i.e. observation smaller than expected).

In contrast to volumes of brain segmentations the morphometry pipeline yields nine parameters per surface parcellation. For comparison with expert rating of morphometric abnormalities and accuracy assessment we condensed them into a single statement per cortex region. Asymmetry indices were counted half for each hemisphere. To suppress the influence of potentially artifactual measurements we calculated a weighted average using the odds for valid vs. artifactual measurement^32^ as weights

2 $${{\rm{L}}}_{{\rm{r}}}=\sqrt{\frac{\sum _{{\rm{m}}}{{{\rm{L}}}_{{\rm{r}}{\rm{m}}}^{2}{{\rm{o}}{\rm{d}}{\rm{d}}{\rm{s}}}_{{\rm{r}}{\rm{m}}}}^{2}}{\sum _{{\rm{m}}}{{{\rm{o}}{\rm{d}}{\rm{d}}{\rm{s}}}_{{\rm{r}}{\rm{m}}}}^{2}}}\ge 0$$

### Selection of subtype-specific features

The full feature vectors may contain many brain regions and morphometric parameters that are rather unspecific for temporal lobe epilepsies. To explore whether feature vectors L_rm_ of equation (1) could discriminate TLE subtypes, we evaluated the performance gain expected by filtering out unrepresentative features with low specificity and discriminative power. In a heuristic exploration we employed two principles. Features were required to be (i) highly *reproducible* within the TLE subtype of interest and (ii) highly *unique* in the sense that they differentiated between the subtype of interest and all other subtypes.

To assess reproducibility, we searched features where the absolute value of the subtype-wise mean in units of the standard deviation was extraordinarily large. As thresholds the upper and lower quartile ±1.5 inter quartile ranges were used, respectively. To estimate uniqueness, we checked which features had significantly different mean between the TLE subtype of interest and all other subtypes (p < 0.01). Finally, the intersection of both feature sets was used as heuristic feature selection.

### Accuracy assessment and statistical testing

To quantify the overall accuracy of automated detections with L_r_ ≥ 2 according to the human rater, we used the diagnostic odds ratio (DOR)^56^ defined as the fraction LR + /LR- of the positive to the negative likelihood ratio. LR + is the ratio of probabilities that the morphometry tool correctly detects an abnormality to the tool erroneously claiming a detection if the structure is in fact normal. Conversely, LR- is the ratio of probabilities that the morphometry tool erroneously judges a structure as normal when it is in fact abnormal to a correct normal classification. Important advantages of the DOR are its independence from the prevalence of abnormal structures and the approximately normal distribution of its logarithm. As secondary accuracy statistics, we calculated the positive predictive value (PPV) and the negative predictive value (NPV), yielding (prevalence-dependent) indicators for the fraction of correctly classified abnormal/normal brain regions, respectively.

Throughout the paper between-group differences were assessed by sequential testing. For ordinal data, 1st level testing of the medians was performed using the nonparametric Kruskal-Wallis test. If the differences between all TLE subtypes were at least marginally significant (p < 0.05) the nonparametric Mann-Whitney-Wilcoxon U-test was used in a pairwise manner as a 2nd level test to reveal which subsets differed. For categorial data randomization tests with N = 10,000 resamplings were used for 1st and 2nd level testing of distributional differences. We always tested whether quantifiers depended on the TLE subtype. For brevity we discuss only significant differences in the main text.

### Ethics statement

This study was approved by the Ethics Committee of the Kanton of Bern.

## Electronic supplementary material

Supplementary Information
