# Publisher Correction: Personalized structural image analysis in patients with temporal lobe epilepsy

**DOI:** 10.1038/s41598-017-16213-8

**Published:** 2018-01-09

**Authors:** Christian Rummel, Nedelina Slavova, Andrea Seiler, Eugenio Abela, Martinus Hauf, Yuliya Burren, Christian Weisstanner, Serge Vulliemoz, Margitta Seeck, Kaspar Schindler, Roland Wiest

**Affiliations:** 1 Support Center for Advanced Neuroimaging (SCAN), University Institute for Diagnostic and Interventional Neuroradiology, Inselspital Bern, University of Bern, Bern, Switzerland; 2 Sleep-Wake- Epilepsy-Center, Department of Neurology, Inselspital Bern, University of Bern, Bern, Switzerland; 3 Epilepsy Clinic, Tschugg, Switzerland; 4 University Hospital of Psychiatry, University of Bern, Bern, Switzerland; 5 Presurgical Epilepsy Evaluation Unit, Neurology Department, University Hospital of Geneva, Geneva, Switzerland

Correction to: *Scientific Reports* 10.1038/s41598-017-10707-1, published online 07 September 2017

The original version of this Article contained extensive errors in the Reference list. References 18–21 were incorrectly listed as references 53–56 and references 22–56 were incorrectly listed as references 18–52 respectively.

In addition, this Article contained the following typographical errors in the reference citations.

In the Introduction section,

“While some authors demonstrated an additional yield of voxel based morphometry (VBM) for analysis^2,18–20^, others stressed the poor sensitivity and specificity of morphometric grey matter (GM) alterations^7,25^”.

now reads:

“While some authors demonstrated an additional yield of voxel based morphometry (VBM) for analysis^2,18–24^, others stressed the poor sensitivity and specificity of morphometric grey matter (GM) alterations^7,25^”.

In the Conclusion section,

“A similar approach with VBM volumes has previously been followed by Huppertz *et al*.^2,14,15^”.

now reads:

“A similar approach with VBM volumes has previously been followed by Huppertz *et al*.^2,18,19^”.

In the Materials and Methods section, under the subsection ‘Automated image procession and quality control’,

“The total volumes of CSF, GM and WM were estimated using FSL (http://fsl.fmrib.ox.ac.uk/fsl/fslwiki/, version 5.0^29^)”.

now reads:

“The total volumes of CSF, GM and WM were estimated using FSL (http://fsl.fmrib.ox.ac.uk/fsl/fslwiki/, version 5.0^33^)”.

“Finally, this article contained an error in Ref.^32^ where”

“Rummel, C. *et al*. A fully automatic pipeline for surface-based morphometry in individual patients. under review (2017)”.

now reads:

“Rummel, C. *et al*. A fully automated pipeline for normative atrophy in patients with neurodegenerative disease. under review at Frontiers in Neurology (2017)”.

