# Supplementary material for: Personalized structural image analysis in patients with temporal lobe epilepsy
Source: Sci Rep. 2017 Sep 7;7:10883. doi: 10.1038/s41598-017-10707-1 (PMC5589799; doi:10.1038/s41598-017-10707-1)
Supplement: Supplementary file 1 — Supplementary Information [file 41598_2017_10707_MOESM1_ESM.pdf]

Supplementary Information for

Personalized structural image analysis in patients with temporal lobe epilepsy

Christian Rummel[1+*], Nedelina Slavova[1*], Andrea Seiler[1,2], Eugenio Abela[1,2], Martinus Hauf[1,3], Yuliya Burren[1,4], Christian Weisstanner[1], Serge Vulliemoz[5], Margitta Seeck[5], Kaspar Schindler[2**], Roland Wiest[1**]

[1]Support Center for Advanced Neuroimaging (SCAN), University Institute for Diagnostic and Interventional Neuroradiology, Inselspital Bern, Switzerland

[2]Department of Neurology, Inselspital Bern, Switzerland

[3]Epilepsy Clinic, Tschugg, Switzerland

[4]University Hospital of Psychiatry, University of Bern, Switzerland

[5]Presurgical Epilepsy Evaluation Unit, Neurology Department, University Hospital of Geneva, Geneva, Switzerland.

[*]C.R. and N.S. contributed equally to the study. [**]K.S. and R.W. share senior authorship.

[+]corresponding author: Christian Rummel (PhD)

Support Center for Advanced Neuroimaging (SCAN), University Institute for Diagnostic and Interventional Neuroradiology, Inselspital, 3010 Bern, Switzerland

Tel. 0041 31 6328038, Fax 0041 31 6324872, crummel@web.de

Supplementary tables

Table S1 (part 1 of 4):

MRI dataset	patient	syndrome	hemisphere	age [years]	sex	disease duration [years]	MR scanner	acquisition sequence	number of exact matches
**MTLE-HS left**									
**P001**	1	MTLE-HS	left	26	m	25	Trio	MDEFT	50
**P002**	1	MTLE-HS	left	26	m	25	Trio	MPR ADNI	0
**P003**	2	MTLE-HS	left	54	f	40	Verio	MPR ADNI	27
**P004**	2	MTLE-HS	left	55	f	41	Trio	MDEFT	40
**P005**	2	MTLE-HS	left	55	f	41	Trio	standard MPR	35
**P006**	3	MTLE-HS	left	34	m	8	Trio	MPR ADNI	0
**P007**	4	MTLE-HS	left	65	f	57	Trio	standard MPR	35
**P008**	5	MTLE-HS	left	18	f	15	Verio	MDEFT	50
**P009**	5	MTLE-HS	left	18	f	15	Trio	standard MPR	35
**P010**	6	MTLE-HS	left	56	f	55	Trio	standard MPR	35
**P011**	6	MTLE-HS	left	57	f	56	Verio	standard MPR	0
**P012**	7	MTLE-HS	left	33	f	30	Trio	MDEFT	40
**P013**	8	MTLE-HS	left	18	f	13	Trio	standard MPR	35
**P014**	9	MTLE-HS	left	45	m	14	Verio	MPR ADNI	26

Patient and MRI characteristics.
Significant group differences ($p < 0.01$) are shown in bold face and trends ($p < 0.05$) by italics.
Abbreviations: ADNI, Alzheimer’s Disease Neuroimaging Initiative; LTLE, lateral temporal lobe epilepsy; MDEFT, modified driven equilibrium transform MRI sequence; MTLE-HS, mesial temporal lobe epilepsy with hippocampal sclerosis; MPR, multi-planar reconstructed MRI sequence; m/f, male/female.

Table S1 (part 2 of 4, caption see p. 2):

MRI dataset	patient	syndrome	hemisphere	age [years]	sex	disease duration [years]	MR scanner	acquisition sequence	number of exact matches
**P015**	10	MTLE-HS	left	67	m	--	Trio	standard MPR	28
**P016**	11	MTLE-HS	left	76	f	--	Trio	standard MPR	35
**P017**	12	MTLE-HS	left	68	m	53	Trio	standard MPR	28
**P018**	13	MTLE-HS	left	28	m	25	Trio	standard MPR	28
**median**				49.5		27.5			35
**IQR**				30.25		29			7.75
**MTLE-HS right**									
**P019**	14	MTLE-HS	right	53	f	49	Trio	standard MPR	35
**P020**	15	MTLE-HS	right	48	m	40	Trio	standard MPR	28
**P021**	16	MTLE-HS	right	54	f	2	Trio	standard MPR	35
**P022**	17	MTLE-HS	right	32	f	12	Trio	standard MPR	35
**P023**	18	MTLE-HS	right	36	f	35	Trio	standard MPR	35
**P024**	19	MTLE-HS	right	30	m	14	Trio	standard MPR	28
**P025**	20	MTLE-HS	right	37	f	--	Verio	MPR ADNI	27
**P026**	21	MTLE-HS	right	47	m	17	Trio	MDEFT	50
**P027**	22	MTLE-HS	right	26	f	26	Trio	standard MPR	35
**P028**	23	MTLE-HS	right	44	m	4	Verio	standard MPR	0
**median**				40.5		17			35
**IQR**				14.75		23			7

Table S1 (part 3 of 4, caption see p. 2):

MRI dataset	patient	syndrome	hemisphere	age [years]	sex	disease duration [years]	MR scanner	acquisition sequence	number of exact matches
**LTLE left**									
**P029**	24	LTLE	left	36	f	16	Trio	standard MPR	35
**P030**	25	LTLE	left	26	f	13	Trio	standard MPR	35
**P031**	25	LTLE	left	25	f	13	Verio	MPR ADNI	27
**P032**	26	LTLE	left	26	m	16	Trio	standard MPR	28
**P033**	27	LTLE	left	31	m	27	Trio	standard MPR	28
**P034**	27	LTLE	left	31	m	27	Trio	standard MPR	28
**P035**	27	LTLE	left	30	m	26	Trio	standard MPR	28
**P036**	28	LTLE	left	21	m	--	Trio	standard MPR	28
**P037**	29	LTLE	left	44	f	36	Trio	MDEFT	50
**P038**	30	LTLE	left	53	f	--	Trio	standard MPR	40
**median**				30.5		21			28
**IQR**				8.75		11.75			7

Table S1 (part 4 of 4, caption see p. 2):

MRI dataset	patient	syndrome	hemisphere	age [years]	sex	disease duration [years]	MR scanner	acquisition sequence	number of exact matches
**LTLE right**									
**P039**	31	LTLE	right	36	m	30	Trio	standard MPR	28
**P040**	32	LTLE	right	24	f	4	Trio	standard MPR	35
**P041**	32	LTLE	right	26	f	6	Trio	standard MPR	35
**P042**	33	LTLE	right	22	m	2	Trio	standard MPR	28
**P043**	33	LTLE	right	22	m	2	Trio	MDEFT	31
**P044**	34	LTLE	right	21	f	--	Trio	standard MPR	35
**P045**	35	LTLE	right	27	m	22	Trio	MPR ADNI	0
**P046**	36	LTLE	right	22	m	--	Trio	standard MPR	28
**P047**	37	LTLE	right	35	f	--	Trio	standard MPR	35
**median**				24		5			31
**IQR**				5		15.5			7
**TLE subtype comparison**									
**1st level test**				*p = 0.030 (Kruskal-Wallis test)*	p = 0.940 (chi2 test)	p = 0.055 (Kruskal-Wallis test)	p = 0.308 (chi2 test)	p = 0.798 (chi2 test)	p = 0.981 (Kruskal-Wallis test)
**2nd level test**				**right LTLE subgroup younger than both MTLE-HS subgroups (p<0.01)**	n.a.	n.a.	n.a.	n.a.	n.a.

Table S2 (part 1 of 9):

MRI data set	patient	number of significant detections	main morphometric MRI findings (three largest peaks in odds-weighted feature vector)	main expert MRI finding	epilepsy surgery: resection site and post-surgical seizure control	main expert finding in routine interictal EEG
**MTLE-HS left**						
**P001**	1	48	***asymmetry Hippocampus 6.2***<br>*left S_oc-temp_med_and_Lingual 5.1*<br>*left S_circular_insula_ant 5.1*<br>left G_cingul-Post-ventral 5.1<br>*left G_parietal_sup 5.1*<br>*left G_insular_short 5.1*<br>*left G_temporal_inf 5.1*<br>left S_calcarine 5.1<br>asymmetry G_front_inf-Opercular 5.1<br>asymmetry G_cuneus 5.1<br>right S_oc_sup_and_transversal 5.1<br>right G_occipital_sup 5.1<br>right S_parieto_occipital 5.1	*HS on the left* (reduced volume, increased signal intensity on T2w / FLAIR images), increased CSF volume with colpocephaly, bilateral perisylvian polymicrogyria	selective amygdalo-hippocampectomy on the left<br>Engel IIb<br>follow-up 2 months	FS and ED fronto-temporo-parietal left
**P002**	1	23	**Left-Lateral-Ventricle 7.6**<br>**Right-Lateral-Ventricle 6.5**<br>**4th-Ventricle 4.5**<br>***asymmetry Hippocampus 2.7***			

Diagnostic characteristics.
The volume segmentations and surface parcellations of the three most significant morphometric abnormalities according to the odds weighted feature vector of equation (2) of the main text are listed together with their value L_r. Agreement with expert MRI rating is indicated by boldface and agreement with expert EEG inspection with italic typesetting. Insignificant abnormalities are enclosed in brackets. If the top 3 abnormalities were in disagreement with both expert MRI rating and expert EEG inspection, in addition the concordant abnormalities with largest $L_r \geq 2$ are listed despite lower ranking.
Abbreviations: CSF, cerebro-spinal fluid; ED, epileptiform discharge; FLAIR, fluid-attenuated inversion recovery MRI sequence; FS, focal slowing; HS, hippocampal sclerosis; ICV, intracranial volume; MTA, medial temporal atrophy score; T2w, T2-weighted imaging; WM, white matter. G/S, gyrus/sulcus.

Table S2 (part 2 of 9, caption on p. 6):

MRI data set	patient	number of significant detections	main morphometric MRI findings (three largest peaks in odds-weighted feature vector)	main expert MRI finding	epilepsy surgery: resection site and post-surgical seizure control	main expert finding in routine interictal EEG
**P003**	2	15	**asymmetry Hippocampus 11.8**<br>asymmetry Pallidum 11.8<br>**asymmetry Amygdala 8.4**			
**P004**	2	8	asymmetry Caudate 8.1<br>**asymmetry Hippocampus 7.9**<br>**asymmetry Amygdala 4.0**	HS on the left (reduced volumes of the left hippocampus and amygdala, increased signal intensity of the left hippocampus on T2w / FLAIR images)	selective hippocampectomy on the left<br>Engel IIb<br>follow-up 14 months	bitemporal FS
**P005**	2	18	asymmetry Pallidum 15.9<br>**asymmetry Hippocampus 14.3**<br>asymmetry Putamen 7.9			
**P006**	3	34	***asymmetry Hippocampus 7.0***<br>increased WM-hypointensities 7.0<br>left S_circular_insula_inf 4.4<br>left S_cingul-Marginalis 4.4<br>left G_front_inf-Orbital 4.4<br>right G_and_S_cingul-Mid-Post 4.4	Reduced WM volume, increased CSF volume and ventricle sizes with colpocephalic configuration, *HS on the left* (reduced hippocampal volume, slightly increased signal intensity on T2w / FLAIR images)	no surgery	bitemporal FS, ED temporal left
**P007**	4	15	***asymmetry Hippocampus 12.5***<br>CSF volume increased 3.3<br>brain volume decreased 3.3	*HS on the left* (reduced volume, increased signal intensity on T2w / FLAIR images)	selective amygdalo-hippocampectomy on the left<br>Engel Ia<br>follow-up 40 months	FS and ED temporal left
**P008**	5	19	ICV volume 15.7<br>***asymmetry Hippocampus 15.7***<br>asymmetry Thalamus-Proper 4.3	*cavernoma on the left mesiotemporal lobe* (popcorn-like, hypointense on T2w images), *reduced hippocampal volume on the left*, old lacunar infarction in the left putamen with volume reduction of the lentiform nucleus; some WM changes	no surgery	FS temporal left
**P009**	5	16	ICV volume 5.7<br>asymmetry Pallidum 5.7<br>non-WM-hypointensities 3.8			

Table S2 (part 3 of 9, caption on p. 6):

MRI data set	patient	number of significant detections	main morphometric MRI findings (three largest peaks in odds-weighted feature vector)	main expert MRI finding	epilepsy surgery: resection site and post-surgical seizure control	main expert finding in routine interictal EEG
**P010**	6	10	ICV volume 8.7<br>***asymmery Hippocampus 8.7***<br>CSF volume 2.7<br>brain volume 2.7	*HS on the left* (reduced volume, increased signal intensity on T2w / FLAIR images)	selective amygdalo-hippocampectomy and resection of temporal pole on the left<br>Engel Ia<br>follow-up 36 months	FS temporal left
**P011**	6	14	***asymmetry Hippocampus 9.1***<br>left S_calcarine 4.4<br>left S_precentral-inf-part 4.4<br>left S_intrapariet_and_P_trans 4.4<br>left S_occipital_ant 4.4<br>right S_occipital_ant 4.4			
**P012**	7	2	Left-Accumbens-area volume 3.4<br>Right-Accumbens-area volume 2.8<br>(right G_temp_sup-G_T_transv 1.9)	Discrete increase of the signal intensity of the *left hippocampus* without significant visual volume reduction	selective amygdalo-hippocampectomy on the left<br>no follow-up	ED temporal left
**P013**	8	8	ICV volume 9.4<br>***asymmetry Hippocampus 9.4***<br>***Left-Hippocampus 2.7***	*HS on the left* (reduced volume, increased signal intensity on T2w / FLAIR images). Suspicion for dual pathology - slight hyperintensity of the middle / inferior temporal gyrus on T2w / FLAIR images)	anterior temporal lobectomy on the left<br>Engel Ia<br>follow-up 42 months	FS and ED fronto-temporal left
**P014**	9	6	asymmetry Thalamus-Proper 7.3<br>asymmetry Putamen 7.1<br>**asymmetry Hippocampus 3.0**	HS on the left (reduced hippocampal volume, widened temporal horn, increased signal intensity of the hippocampus and the parahippocampal gyrus on T2w / FLAIR images)	no surgery	normal
**P015**	10	12	***asymmetry Hippocampus 6.1***<br>left G_occipital_middle 2.7<br>right S_front_sup 2.7<br>right G_and_S_occipital_inf 2.7<br>right S_temporal_sup 2.7<br>right G_and_S_cingul-Mid-Post 2.7	*HS on the left* (reduced volume of the left hippocampus). Old (post-traumatic) parenchymal defect of the left frontal lobe	no surgery	ED fronto-temporal left

Table S2 (part 4 of 9, caption on p. 6):

MRI data set	patient	number of significant detections	main morphometric MRI findings (three largest peaks in odds-weighted feature vector)	main expert MRI finding	epilepsy surgery: resection site and post-surgical seizure control	main expert finding in routine interictal EEG
**P016**	11	63	left S_oc_middle_and_Lunatus 10.0<br>left S_oc-temp_lat 10.0<br>left S_oc_sup_and_transversal 10.0<br>left G_and_S_frontomargin 10.0<br>left S_circular_insula_sup 10.0<br>left Lat_Fis-post 10.0<br>left G_front_sup 10.0<br>left S_precentral-inf-part 10.0<br>left G_pariet_inf-Supramar 10.0<br>left G_and_S_subcentral 10.0<br>left S_collat_transv_post 10.0<br>left S_front_inf 10.0<br>right S_oc_sup_and_transversal 10.0<br>right S_circular_insula_sup 10.0<br>right G_and_S_cingul-Mid-Ant 10.0<br>right S_subparietal 10.0<br>right G_and_S_subcentral 10.0<br>right S_circular_insula_ant 10.0<br>right S_temporal_sup 10.0<br>right G_precentral 10.0<br>**right S_front_sup 10.0**<br>right G_and_S_cingul-Ant 10.0<br>right S_oc-temp_med_and_Lingual 10.0<br>right S_orbital-H_Shaped 10.0<br>right S_temporal_inf 10.0<br>right Lat_Fis-ant-Horizont 10.0<br>right Lat_Fis-ant-Vertical 10.0<br>right S_occipital_ant 10.0<br>right G_oc-temp_lat-fusifor 10.0<br>**right S_front_middle 3.5**<br>**right S_front_inf 3.2**<br>**right G_front_middle 2.2**<br>**asymmetry Hippocampus 2.2**	*HS on the left* (reduced hippocampal volume, increased signal intensity on T2w / FLAIR images), many white matter lesions. Suspicion for focal cortical dysplasia of the right middle superior gurys (frontal lobe)	no surgery	bitemporal FS, enhanced on the left
**P017**	12	45	***Right-Inf-Lat-Vent 14.1***<br>***asymmetry Hippocamus 14.1***<br>**3rd-Ventricle 8.4**	Global cortical atrophy, increased CSF volume, *bilaterally reduced hippocampal volumes*, marked HS on the left (MTA 4)	no surgery	bitemporal FS, enhanced on the right
**P018**	13	3	asymmetry Pallidum 2.1<br>**Left-Cerebellum-Cortex 2.0**<br>***Right-Hippocampus 2.0***	HS on both sides (slightly reduced hippocampal volumes, increased signal intensity of the hippocampus on T2w / FLAIR images, mainly on the left). Moderate cerebellar atrophy	no surgery	FS temporal right

Table S2 (part 5 of 9, caption on p. 6):

MRI data set	patient	number of significant detections	main morphometric MRI findings (three largest peaks in odds-weighted feature vector)	main expert MRI finding	epilepsy surgery: resection site and post-surgical seizure control	main expert finding in routine interictal EEG
**MTLE-HS right**						
**P019**	14	excluded	n.a.	*HS on the right* (slightly reduced volume, increased signal intensity on T2w / FLAIR images)	no surgery	FS fronto-temporal right
**P020**	15	excluded	n.a.	*HS on the right* (reduced volume, increased signal intensity on T2w / FLAIR images)	no surgery	FS and ED fronto-temporal right
**P021**	16	excluded	n.a.	*HS on the right* (marked signal increase on T2w / FLAIR images without volume reduction)	amygdala-uncus-hippocampectomy on the right<br>Engel Ia<br>follow-up 4 months	FS and ED fronto-temporal right
**P022**	17	3	ICV 6.7<br>***asymmetry Hippocampus 6.7***<br>right G_precuneus 2.0	*HS on the right* (reduced volume, increased signal intensity on T2w / FLAIR images)	selective amygdalo-hippocampectomy on the right<br>Engel Ia<br>follow-up 28 months	FS temporal right
**P023**	18	8	***asymmetry Hippocampus 5.2***<br>left Lat_Fis-post 3.4<br>left S_front_inf 3.4<br>right S_central 3.4<br>right G_cingul-Post-ventral 3.4	*HS on the right* (markedly reduced volume, increased signal intensity on T2w / FLAIR images)	no surgery	ED temporal right
**P024**	19	excluded	n.a.	Discreet alteration of the *right hippocampus / HS* (slightly reduced volume, discreetly increased signal intensity on T2w / FLAIR images)	no surgery	FS temporal right
**P025**	20	7	asymmetry Thalamus-Proper 4.4<br>**Left-Amygdala 2.8**<br>asymmetry Pallidum 2.5	Dual pathology: T2w hyperintense mass of the right uncus / amygdala (low-gradie glioma or MCD - malformation of cortical development), in association with HS on the right (reduced volume of the hippocampus)	no surgery	normal
**P026**	21	3	**asymmetry Hippocampus 2.3**<br>left G_occipital_middle 2.1<br>right G_pariet_inf-Supramar 2.1	HS on the right (only slightly reduced volume, hyperintensity on T2w / FLAIR images), alteration of the right praecuneus (discreet subcortical hyperintensity on FLAIR images)	no surgery	n.a.

Table S2 (part 6 of 9, caption on p. 6):

MRI data set	patient	number of significant detections	main morphometric MRI findings (three largest peaks in odds-weighted feature vector)	main expert MRI finding	epilepsy surgery: resection site and post-surgical seizure control	main expert finding in routine interictal EEG
**P027**	22	10	ICV 4.4<br>asymmetry Thalamus-Proper 4.4<br>asymmetry Pallidum 3.9<br>**asymmetry Hippocampus 2.8**	*HS on the right* (reduced volume, increased signal intensity on T2w / FLAIR images)	no surgery	discrete FS temporal left
**P028**	23	14	**asymmetry Hippocampus 5.8**<br>asymmetry Thalamus-Proper 4.5<br>Left-Thalamus-Proper volume 2.6	HS on the right (reduced volume, increased signal intensity on T2w / FLAIR images), discreet volume reduction of the left hippocampus	no surgery	normal
**LTLE left**						
**P029**	24	0	(Right-Lateral-Ventricle volume 1.9<br>left S_circular_insula_sup 1.8<br>left G_oc-temp_med-Lingual 1.8<br>right S_parieto_occipital 1.8)	non-lesional	no surgery	FS temporal left
**P030**	25	8	ICV 4.4<br>asymmetry Pallidum 4.4<br>Left-Pallidum volume 2.9	non-lesional	anterior 2/3 temporal lobectomy on the left<br>Engel Ia<br>follow-up 32 months	bilateral intermittent asynchronous EEG
**P031**	25	10	left G_front_inf-Triangul 4.8<br>left S_pericallosal 4.8<br>left G_Ins_lg_and_S_cent_ins 4.8<br>left S_postcentral 4.8<br>right Lat_Fis-post 4.8<br>right S_parieto_occipital 4.8			
**P032**	26	excluded	n.a.	non-lesional	lateral-anterior temporal lobectomy on the left<br>Engel Ia<br>follow-up 12 months	FS fronto-temporo-parieto-occipital left, ED temporal left

Table S2 (part 7 of 9, caption on p. 6):

MRI data set	patient	number of significant detections	main morphometric MRI findings (three largest peaks in odds-weighted feature vector)	main expert MRI finding	epilepsy surgery: resection site and post-surgical seizure control	main expert finding in routine interictal EEG
**P033**	27	11	asymmetry Pallidum volume 3.7<br>right G_and_S_cingul-Mid-Post 2.6<br>*left S_temporal_transverse 2.6*<br>*asymmetry Pole_temporal 2.6*<br>left S_oc_sup_and_transversal 2.6<br>left G_precuneus 2.6<br>*left G_temp_sup-Plan_tempo 2.2*<br>**Right-Hippocampus volume 2.1**			
**P034**	27	10	asymmetry Thalamus-Proper 6.0<br>left G_pariet_inf-Supramar 2.8<br>left G_precentral 2.8<br>left G_insular_short 2.8<br>left S_oc-temp_lat 2.8<br>right Pole_temporal 2.8<br>right G_and_S_occipital_inf 2.8<br>***Left-Hippocampus volume 2.0***	bilateral HS (volume reduction mainly on the right, bilateral hyperintensity of the hippocampi on T2w / FLAIR images)	no surgery	FS fronto-temporal left
**P035**	27	7	asymmetry Thalamus-Proper 6.5<br>asymmetry Pallidum 4.3<br>Left-Thalamus-Proper volume 3.5			
**P036**	28	5	asymmetry Thalamus-Proper 5.5<br>asymmetry Pallidum 2.9<br>Left-Thalamus-Proper volume 2.3	Dual pathology: *tumor of the temporopolar region on the left* (DNET) and *HS* (discreet signal increase on T2w / FLAIR images)	no surgery	FS fronto-temporal left
**P037**	29	4	**left G_temp_sup-G_T_transv 2.4**<br>left S_circular_insula_inf 2.2<br>left G_cingul-Post-dorsal 2.1	Focal cortical dysplasia type II with subcortical T2w hyperintensity of the superior and middle temporal gyrus on the left, "transmantle sign" up to the lateral ventricle	micro-surgical removal of focal cortical dysplasia on superior temporal gyrus on the left<br>Engel Ic<br>follow-up 4 months	normal

Table S2 (part 8 of 9, caption on p. 6):

MRI data set	patient	number of significant detections	main morphometric MRI findings (three largest peaks in odds-weighted feature vector)	main expert MRI finding	epilepsy surgery: resection site and post-surgical seizure control	main expert finding in routine interictal EEG
**P038**	30	16	asymmetry Thalamus-Proper 3.8<br>3rd-Ventricle volume 2.9<br>*left G_temporal_inf 2.8*<br>left G_cuneus 2.8<br>left G_occipital_sup 2.8<br>left S_front_sup 2.8<br>right S_pericallosal 2.8<br>right S_central 2.8<br>right S_circular_insula_sup 2.8	non-lesional	no surgery	ED temporal left
**LTLE right**						
**P039**	31	8	asymmetry Lateral-Ventricle volume 4.3<br>right S_central 4.0<br>right G_and_S_subcentral 4.0<br>**left S_front_middle 3.1**<br>**left G_and_S_frontomargin 2.9**	Multiple cortical-subcortical signal alterations consistant with tuberous sclerosis (e.g. on the left frontal lobe, middle frontal gyrus on the right, precentral gyrus on the right, supramarginal gyrus on the right, cuneus on the right). Remote cerebellar hemorrhage on the left	no surgery	FS temporal right
**P040**	32	8	ICV 4.0<br>asymmetry Thalamus-Proper 4.0<br>asymmetry Pallidum 3.3	HS on the right - discreet finding - signal increase of the right hippocampus on 3D T2w images	angular and supramarginal gyrus on the right<br>Engel IIa<br>follow-up 31 months	bitemporal FS
**P041**	32	13	left S_calcarine 8.4<br>asymmetry G_and_S_cingul-Mid-Post 8.4<br>right G_and_S_subcentral 8.4<br>right G_front_inf-Opercular 8.4<br>***right G_oc-temp_med-Parahip 8.4***<br>right S_pericallosal 8.4<br>*right S_oc-temp_med_and_Lingual 8.4*			

Table S2 (part 9 of 9, caption on p. 6):

MRI data set	patient	number of significant detections	main morphometric MRI findings (three largest peaks in odds-weighted feature vector)	main expert MRI finding	epilepsy surgery: resection site and post-surgical seizure control	main expert finding in routine interictal EEG
**P042**	33	17	left Pole_occipital 8.4<br>left S_circular_insula_ant 8.4<br>left S_central 8.4<br>left G_cuneus 8.4<br>**asymmetry S_circular_insula_ant 8.4**<br>**right G_front_inf-Orbital 8.4**<br>**right S_circular_insula_ant 8.4**<br>**right G_orbital 3.1**<br>**right G_insular_short 2.3**<br>**asymmetry G_orbital 2.1**	Multiple cavernomas on the right (e.g. in the right precentral gyrus, right anterior insula / frontoorbital gyrus, right lateral occipito-temporal gyrus)	resection of cavernomas on the right<br>Engel Ia<br>follow-up 29 months	normal
**P043**	33	13	**right G_front_inf-Orbital 6.7**<br>right Lat_Fis-ant-Horizont 6.7<br>right S_collat_transv_ant 6.7<br>right G_front_inf-Triangul 6.7<br>asymmetry G_front_inf-Triangul 6.7<br>asymmetry Lat_Fis-ant-Horizont 6.7			
**P044**	34	3	right G_and_S_subcentral 3.5<br>right S_front_middle 3.5<br>asymmetry vessel volume 2.0	non-lesional	no surgery	ED bi-fronto-temporal
**P045**	35	0	(asymmetry S_occipital_ant 1.4<br>asymmetry G_front_sup 1.3<br>asymmetry G_occipital_middle 1.2)	Focal cortical dysplasia on the left (cortical-subcortical T2w / FLAIR hyperintensity around the occipito-temporal sulcus)	no surgery	normal
**P046**	36	2	asymmetry Thalamus-Proper 2.6<br>asymmetry Inf-Lat-Vent 2.0<br>(left G_temp_sup-Plan_polar 1.8<br>asymmetry G_oc-temp_med-Parahip 1.8<br>right Pole_temporal 1.8<br>right S_oc_sup_and_transversal 1.8)	non-lesional	no surgery	ED temporal right
**P047**	37	19	***right G_temporal_inf 5.9***<br>***right S_oc-temp_med_and_Lingual 5.9***<br>right S_collat_transv_ant 5.9<br>***right G_oc-temp_lat-fusifor 5.9***<br>***right S_occipital_ant 5.9***<br>right S_circular_insula_inf 5.9<br>***asymmetry G_temporal_inf 5.9***	*Extensive polymicrogyria of the right temporal and occipital lobe, malrotation of the right hippocampus and HS on the right* (reduced volume and increased signal intensity on T2w / FLAIR images)	no surgery	ED temporal right

Supplementary figures

Figure S1

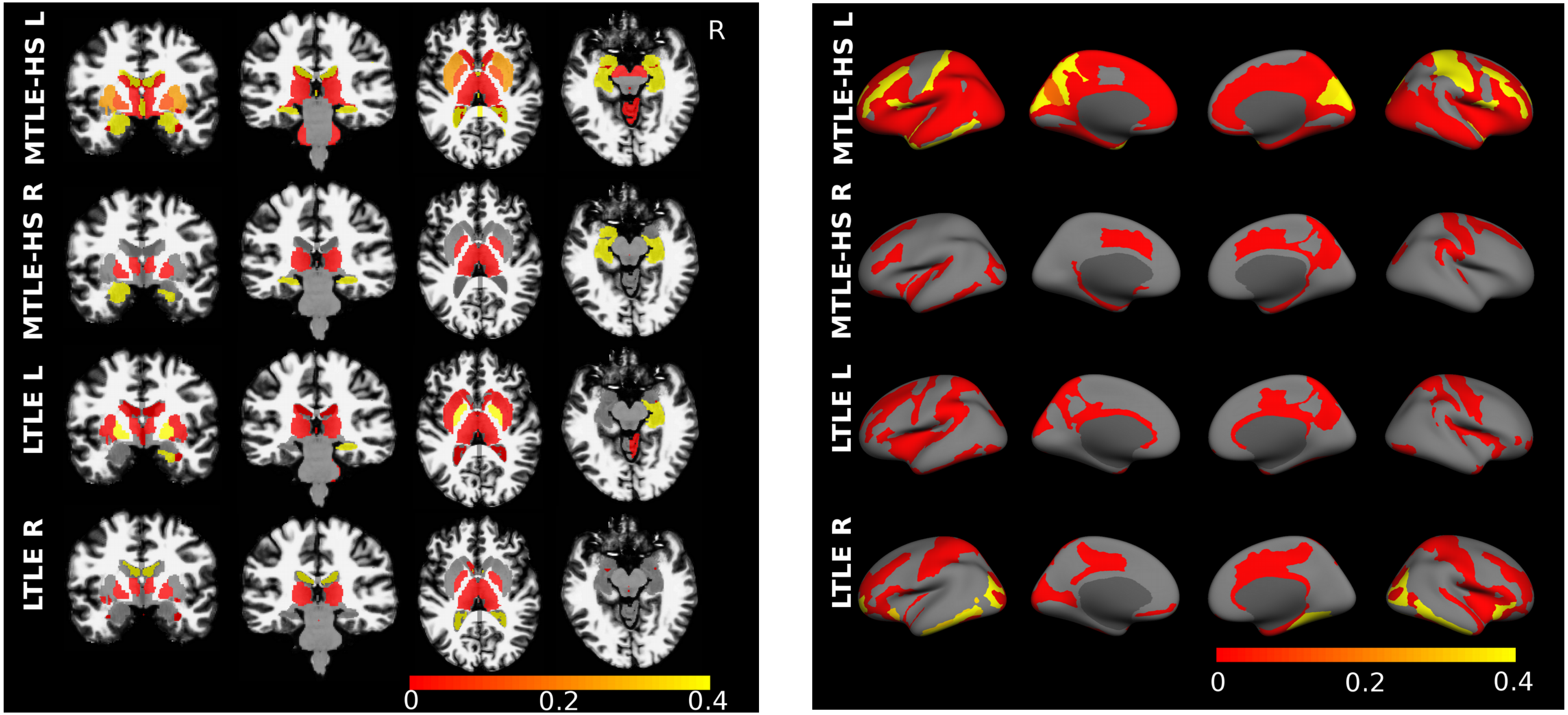

Spatial distribution of positive predictive values (PPV)
Regional PPV were calculated as the percentage of patients in a TLE subtype, where abnormalities detected by the automated morphometry tool were confirmed abnormal by the neuroradiologist (number of true test positives divided by number of all test positives). Volume segmentations and surface parcellations are left grey if neither the automated pipeline nor the expert detected an abnormality. Largest PPV was reached for hippocampal volume loss in the mesial temporal lobe epilepsies with hippocampal sclerosis and for volume loss of the pallidum in the lateral temporal lobe epilepsies with left-sided seizure onset. Surface parcellations with high PPVs were the pre- and postcentral gyri and sulci bilaterally as well as the cuneus in the mesial temporal lobe epilepsies with left-sided seizure onset. In the lateral temporal lobe epilepsies with right-sided seizure onset the highest PPV was observed in the inferior temporal gyri and in the insula bilaterally.

Figure S2

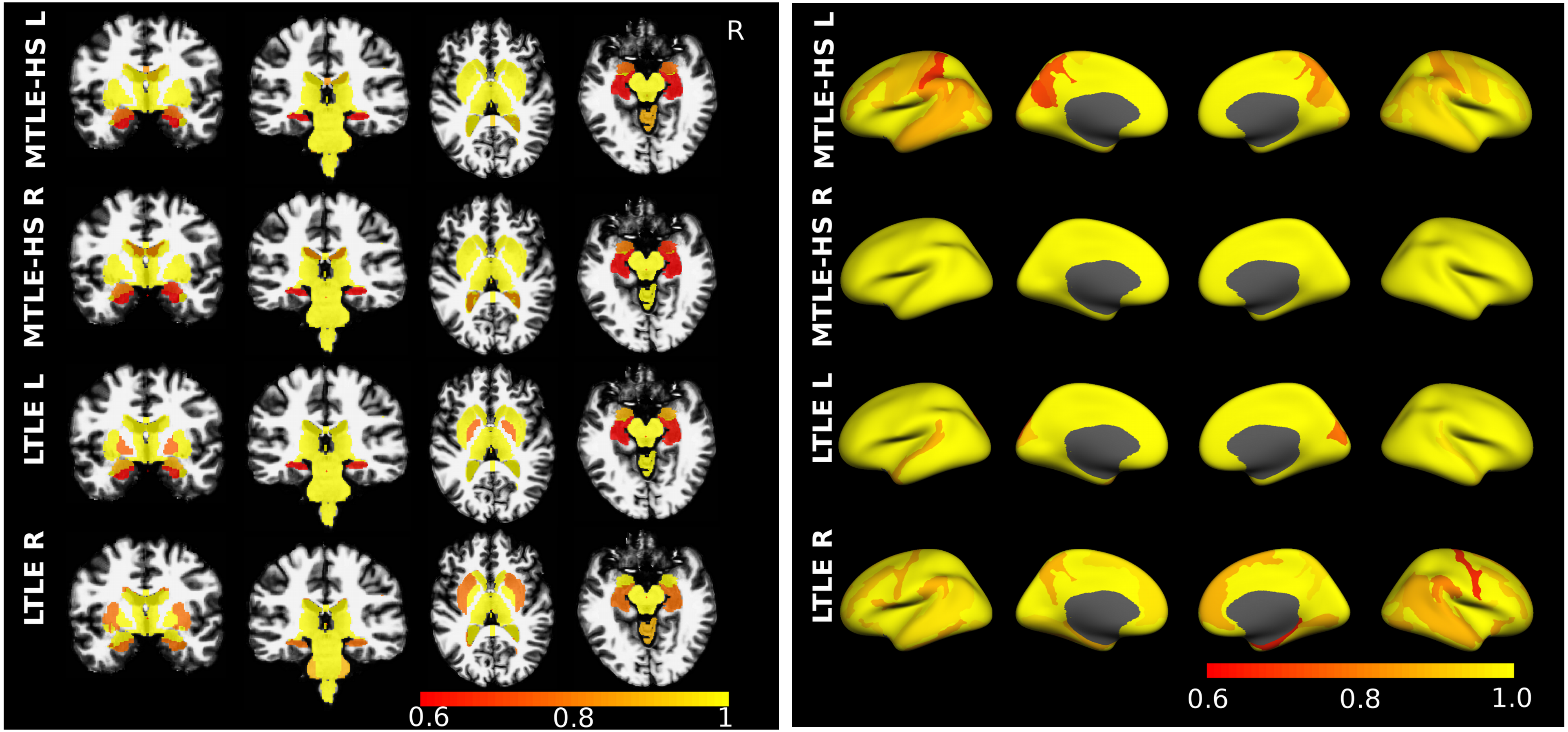

Spatial distribution of negative predictive values (NPV)
Regional NPV were calculated as the percentage of patients in a TLE subtype, where regions rated as normal by the automated morphometry tool were also rated normal by the neuroradiologist (number of true test negatives divided by number of all test negatives). In volume segmentations smallest NPV was observed for hippocampal volume loss in all TLE subtypes, indicating that not all hippocampal scleroses were correctly identified by the automated morphometry pipeline. For surface parcellations NPV was smallest for the left precuneus and bilateral postcentral gyrus in the mesial temporal lobe epilepsies with left-sided seizure onset and in the right precentral gyrus of the lateral temporal lobe epilepsies with right-sided seizure onset.

Figure S3

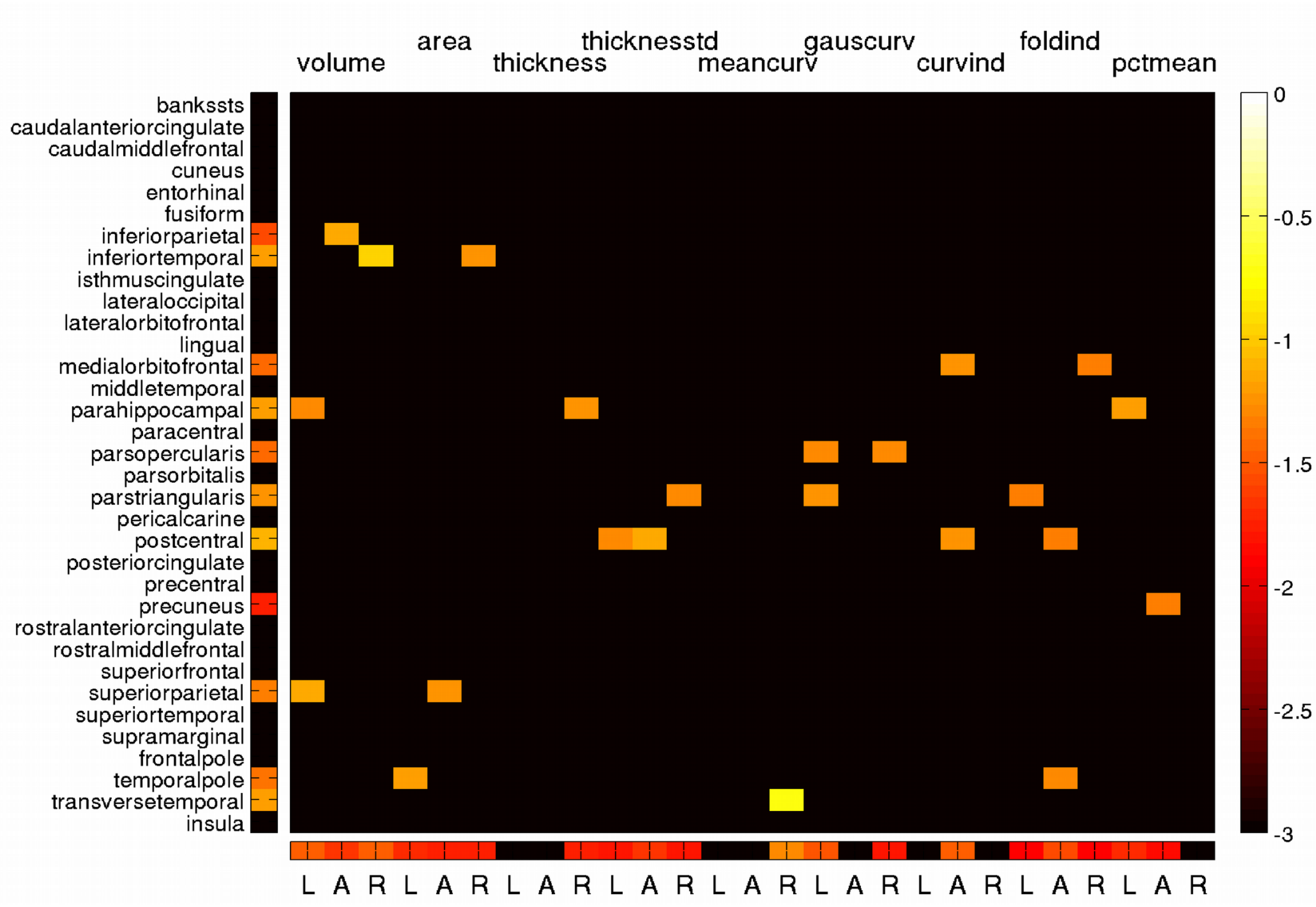

Cortical features of the Desikan-Killiany atlas selected to represent TLE subtypes

Cortical features of the Desikan-Killiany atlas selected to represent at least one of the TLE subtypes. The color coding represents the decadic logarithm of relative feature importances (values for all features add up to one). Abbreviations: A, asymmetry index; L/R, left/right; thicknessstd, standard deviation of cortical thickness; meancurv, mean curvature; gausscurv, Gaussian curvature; curvind, curvature index; foldind, folding index; pctmean, mean percentage change of the grey-white contrast

Figure S4

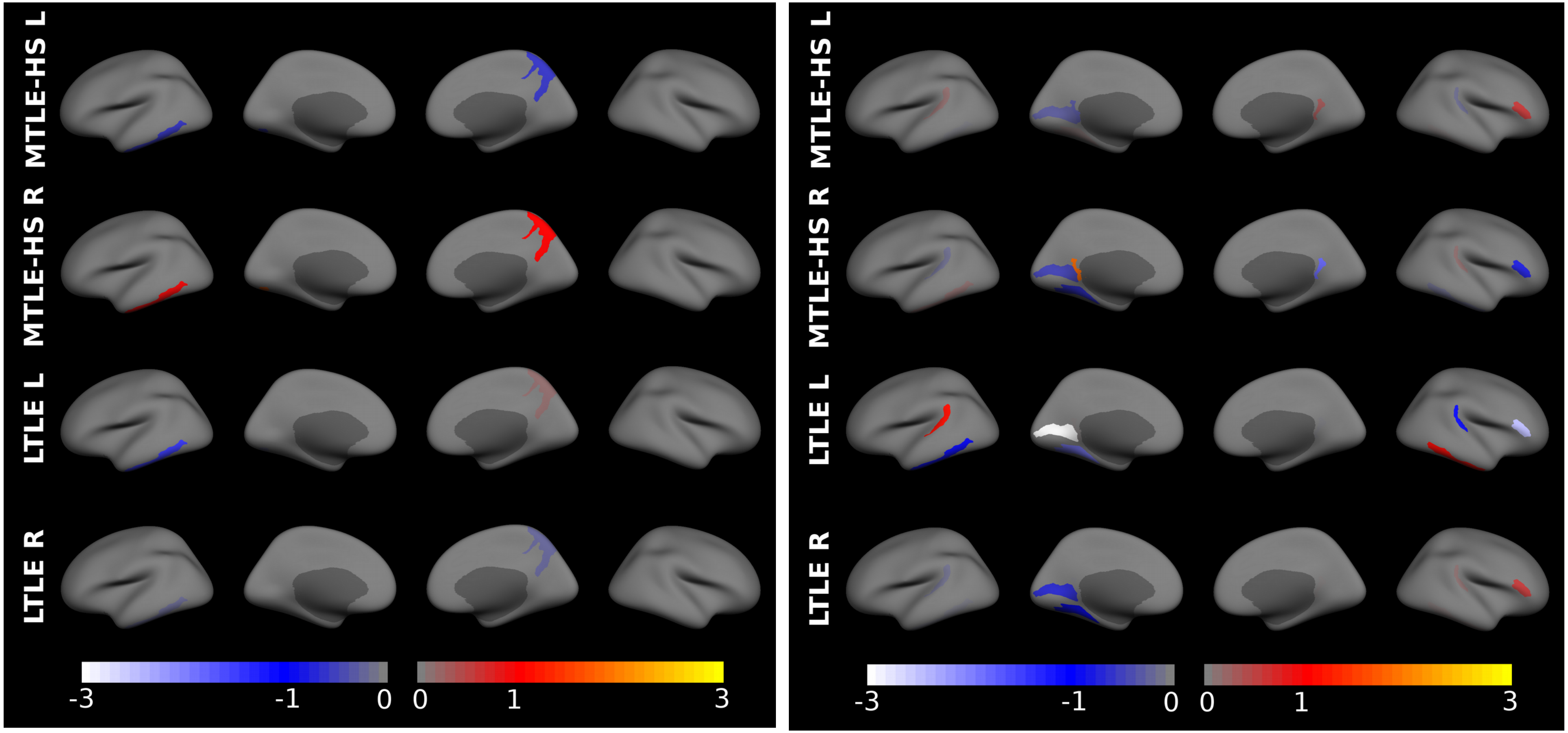

Spatial distribution of features selected to represent TLE subtypes
Subtype-wise z-scores of cortical GM volume (left) and cortical thickness features (right, mean and standard deviation merged).

Figure S5

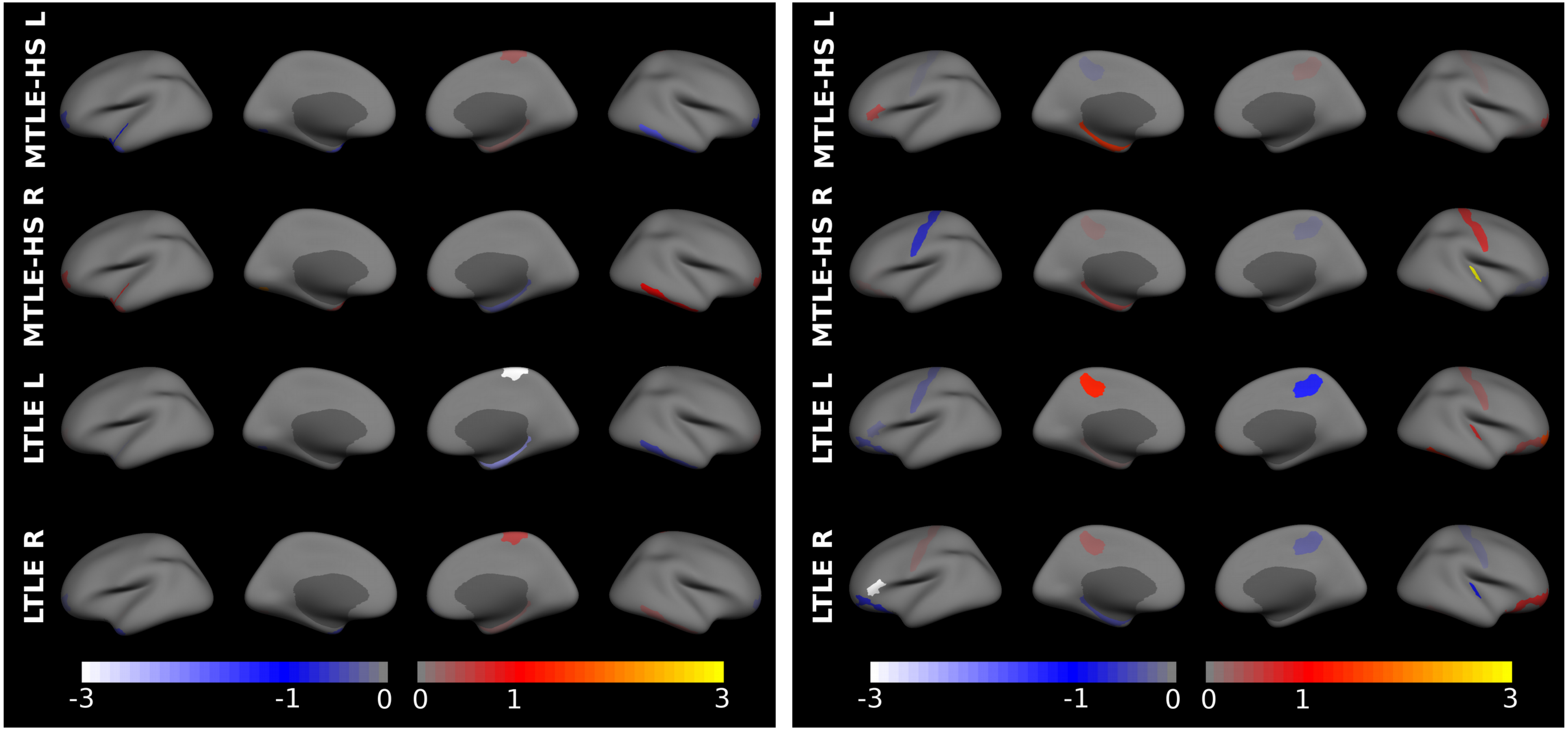

Spatial distribution of features selected to represent TLE subtypes
Subtype-wise z-scores of cortical surface area (left) and cortical curvature features (right, all four curvatures merged).

Figure S6

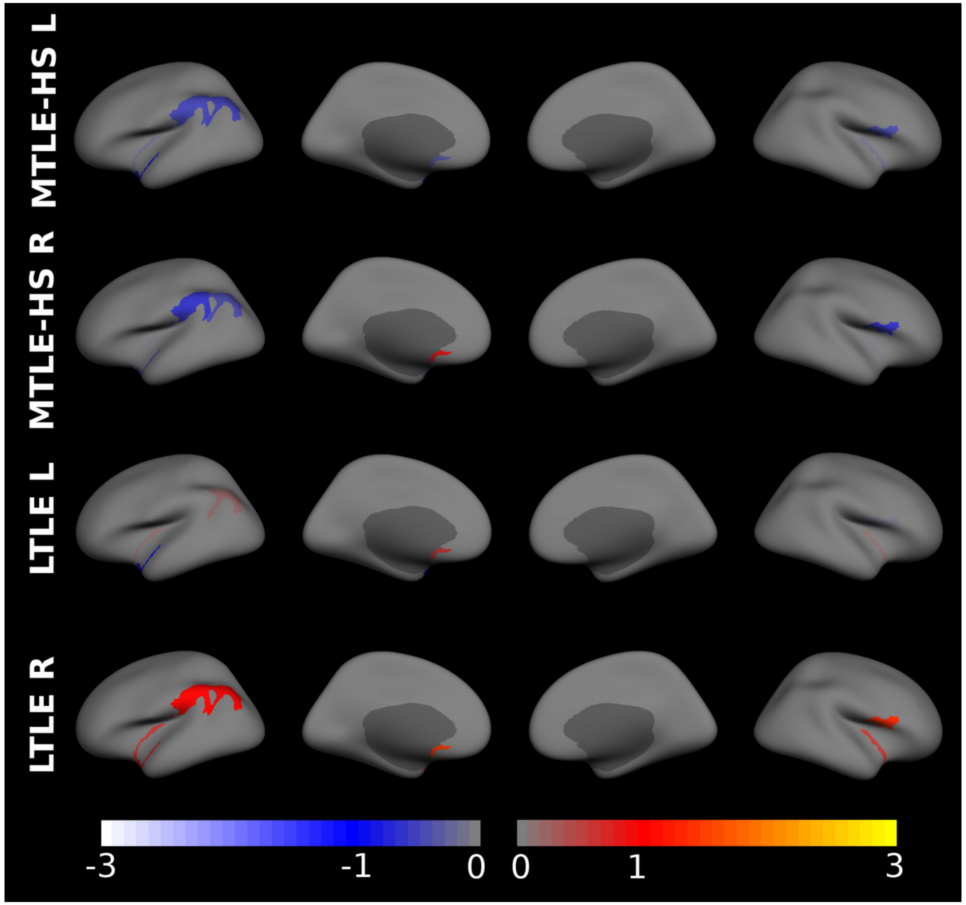

Spatial distribution of features selected to represent TLE subtypes
Subtype-wise z-scores of cortical grey-white contrast features.

Supplementary methods

MR scanners and acquisition sequences

All MR images were acquired on two different 3T MR scanners at the Inselspital Bern (Verio and Magnetom Trio, Siemens, Erlangen/Germany). Every subject underwent a standardized T1-weighted MRI protocol including either 3D MP-RAGE[1] or MDEFT[2] or an MP-RAGE according to the Alzheimer's Disease Neuroimaging Initiative's (ADNI) recommendations[3] in sagittal acquisition and with 1 mm isotropic resolution. Experimentally we also acquired healthy control datasets with an MP-RAGE optimized for grey-white contrast as recommended by van der Kouwe et al.[4]. Detailed sequence parameters are summarized in Table S3.

	**MDEFT[2]**	**MP-RAGE standard[1]**	**MP-RAGE[3]**	**MP-RAGE[4]**
**voxel size (mm)**	1.0*1.0*1.0	1.0*1.0*1.0	1.0*1.0*1.0	1.0*1.0*1.0
**field of view (mm)**	256*224	256*256	256*256	250*250
**slices**	176	160	160	160
**matrix**	256*224*176	256*256*160	256*256*160	250*250*160
**repetition time TR (ms)**	7.92	1500	2300	2530
**echo time TE (ms)**	2.48	2.01	2.98	3.37
**inversion time TI (ms)**	910	900	1100	1100
**flip angle (°)**	16	9	9	7
**fat saturation**	yes	no	no	no
**acquisition time (min)**	13'43”	3'30”	5'21”	10'49”

Table S3:
Basic parameters of the used MRI acquisition sequences.

Expert MRI analysis

The patient MRIs were rated by consensus-reading of a neuroradiologist in training (NS) with a senior specialist with training in neuroimaging of epilepsy with 15 (RW, senior consultant) and seven years (CW, senior physician) of expertise according to the standards of Berkovic et al.[5] and Coan et al.[6] to identify i) FLAIR- and/or T2w hyperintense signals in the hippocampus, amygdala and/or lateral temporal lobe on coronal slices angulated along the hippocampus, ii) volume loss of the hippocampus proper and/or amygdala on high resolution T1-weighted images after axial reslicing along the hippocampal plane and iii) loss of anatomical configuration on either i) or ii). We further aimed to rule out subtle signs of cortical dysplasia, especially abnormal sulcal morphology, local increases of cortical thickness, FLAIR focal signal hyperintensities or subtle transmantle signs.

In patients who underwent epilepsy surgery, also post-surgical follow-up MRIs were rated by the experts. They assessed the resection area and its overlap with volume segmentations and surface parcellations used by the morphometry pipeline for further comparison.

Expert EEG analysis

Routine scalp EEGs with minimal time difference to MRI acquisition were obtained from our clinical data base. Duration of awake EEGs was at least 30 minutes. All EEGs were reviewed independently by a fellow in neurophysiology (AS) and an experienced board-certified epileptologist (KS), establishing a final consensus in accordance with the patient's seizure semiology. Both were blinded to the patients' imaging data. The EEGs were evaluated for presence (yes or no) and localization (left or right hemisphere; lobar localization if possible) of focal slowing and epileptiform signals.

Voxel based volumetry with FSL

The volumes of CSF, GM and WM were estimated using the free software package FSL[7] (`http://fsl.fmrib.ox.ac.uk/fsl/fslwiki/`, version 5.0) developed at the Oxford Centre for Functional MRI of the Brain (FMRIB). First, the images were automatically scull stripped using the Brain Extraction Tool (BET[8], version 2.1) with robust brain center estimation (option `-R`) and automated clean-up for eye and optic nerve contributions (option `-S`) as well as for bias field and neck (option `-B`). The fractional intensity threshold (option `-f`) was set to 0.3. Using FMRIB's Automated Segmentation Tool (FAST[9], version 5.0.6) the brain voxels were then classified into three tissue classes (CSF, GM and WM) with sum of probabilities equal to one. Partial volume estimates for each tissue class were calculated by integrating the voxel-wise tissue class probabilities over the whole volume. In addition to GM, WM and CSF volumes, we calculated the brain volume (i.e. sum of GM and WM volume) and the intracranial volume (ICV, i.e. the sum of GM, WM and CSF volumes).

Volume segmentations by FreeSurfer

Volumes of GM, WM and CSF segmentations were estimated using the free software package FreeSurfer (`https://surfer.nmr.mgh.harvard.edu`, version 5.3.0) developed at the Laboratory for Computational Neuroimaging of the Athinoula A. Martinos Center for Biomedical Imaging. The used procedures are described in detail in Fischl et al.[10,11]. In addition, FreeSurfer’s estimated total intracranial volume (eTIV) was used to isometrically normalize all morphometric parameters for brain size.

We evaluated the reliability of FreeSurfer’s volume estimations assessing the relative measurement error from the eight TLE patients who had repeated MRIs. Relative errors for ICV and eTIV were not different ($p = 0.060$, paired t-test). Despite more patients with seizure onset on the left than on the right had repeated MRI and accuracy might be impaired by pathology[12,13] there was no side difference between the relative errors for hippocampi ($p = 0.374$) and amygdalae ($p = 0.194$).

Surface based morphometry with FreeSurfer

Surface based morphometry was also performed with FreeSurfer. The technical details of these procedures have been described previously in Dale et al.[15] and Fischl et al.[16,17]. The processing includes automated tessellation of the GM-WM interface using vertices and faces. Surfaces were deformed following intensity gradients to optimally place the GM-WM and GM-CSF interfaces at the location where the greatest intensity change defines the transition to the other tissue class.

The following nine morphometric parameters were evaluated at all vertex points of the surfaces and averaged over cortex parcellations: cortical surface area, mean and standard deviation of the cortical thickness, cortical GM volume, mean cortical curvature, Gaussian curvature, curvature index, folding index and grey-white contrast.

- The surface area of a cortex parcellation was estimated by summing up the face areas of all included vertices of the tesselated surface.
- Cortical thickness was calculated with sub-millimeter resolution as the closest distance from the GM-WM interface to the GM-CSF interface at each vertex[18]. Mean and standard deviation of the cortical thickness were evaluated for each parcellation.
- The cortical GM volume was calculated as the parcellation-wise volume enclosed by the GM-WM interface and the GM-CSF interface.
- Curvature and folding measures were defined from the inverse radii of ellipsoids that approximated the surfaces locally. They can either be extrinsic properties of the surface embedded into 3D space or intrinsic properties of the surface itself[19]. The extrinsic mean curvature is given by the mean of the local minimal and maximal curvature (dimension 1/mm), whereas the intrinsic Gaussian curvature is the product of both (dimension $1/mm^2$).
- The grey-white contrast was estimated by extending the GM-WM interface 1 millimeter into the WM and 35% of the local cortical

thickness into the GM according to Salat et al.[20]. At voxel size 1mm this procedure ensures that the target points are located in different voxels and the contrast was estimated as these voxels' intensity difference normalized to their intensity sum. It ranges between 0 for identical voxel intensities and 1 for maximal intensity difference.

Quality control procedures

FreeSurfer's automatic surface tesselation, segmentation and parcellation procedures may occasionally produce errors, which in turn may decrease the accuracy of abnormality detection. False positives are likely to occur when errors are present in the patient dataset, yielding erroneously large values in certain measures. To limit this kind of classification errors in the patient group we assessed the quality of FreeSurfer's procedures using the QA Tools package (`https://surfer.nmr.mgh.harvard.edu/fswiki/QATools`, version 1.1 with adjustments for FreeSurfer version 5.3) and the script `recon_checker`. Morphometric estimates from patient datasets that did not pass these automated and conditional secondary visual quality checks were excluded entirely from further analysis.

In the 323 healthy controls we did not follow these time consuming procedures. Rather we relied on a brain region-wise statistical identification of outliers and excluded them from the normative database. Details are described in Rummel et al.[14].

Standardized result presentation

A standardized result presentation to support expert inspection of T1-weighted MRI was developed in Rummel et al.[14] which we used here without modification. We displayed the patients' morphometric parameters and their region-specific measurement accuracies together with the normative values as a function of age, see Figure S7 for an example. We used an in-house written Octave script (CR) to generate a standardized result display for all morphometric parameters, all volume segmentations and all surface parcellations without preselection. To inform the user about statistically significant deviations from the healthy controls, the panel background was conditionally colored in yellow (p < 0.01, uncorrected) or red (false discovery rate corrected). All results were automatically stored as png figures and html pages to allow unrestricted navigation and switching between region-based and measure-based result compilations.

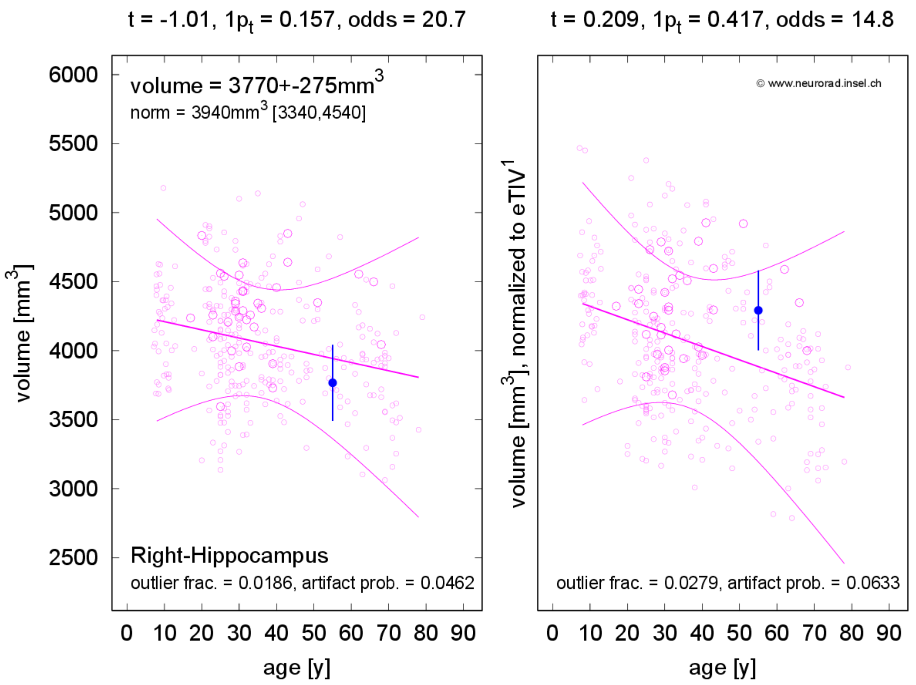

Figure S7:
Standardized result display of morphometric parameters as a function of age using the example of the volume of the non-atrophic right hippocampus of **P005**, see also Figure 1 of the main text. The patient's parameter estimates are shown as a filled symbol with error bars representing the estimated measurement reliability. The open symbols represent the values for the healthy controls. Large open symbols are controls matching the patient exactly for sex, MR scanner and acquisition sequence; statistics is restricted to this subset. Small open symbols have at least one mismatch and are not considered for statistics. The best-fitting polynomial age trend[14] and its confidence bounds are shown as solid lines. The left sub-panel reports parameter values in physical units, whereas normalized values are shown in the right sub-panel. At the top of each panel the test statistics and their significance are reported together with the odds for a valid versus erroneous measurement[14]. At the bottom of each panel the empirical outlier fraction and the estimated artifact probability are reported.

Properties of screening tests

Some diseases have devastating course if untreated but good chance for successful cure or disease modification if treated early on. In such cases broadly used screening tests[21,22,23] are useful. Their statistical properties can be formulated in terms of positive and negative predictive values. PPV is the probability that a patient has the disease if the test is positive. Similarly, NPV is the probability that a patient does not have the disease if the test is negative. For screening tests one typically demands high NPV, meaning that the probability 1-NPV to miss a diagnosis when the test is negative is very low. In contrast, one often accepts moderate (or even low) PPV, because positive screening tests can be followed up with subsequent tests.

For example, a recent review on screening tests for gestional diabetis mellitus by Donovan et al.[24] reported sensitivities and specificities for a variety of tests in their Table 2. Assuming a mean prevalence of 8.7% as suggested by De Sisto et al.[25] one can calculate the mean PPV as 0.314 (range 0.090 to 1.000) and the mean NPV as 0.968 (range 0.920 to 0.999). With a few exceptions the PPVs and NPVs of our morphometry tool shared these properties (overall means were 0.113 for PPV and 0.974 for NPV), see Figures S1 and S2.

Supplementary references

1. Held, P., Fellner, C., Fellner, F., Geissler, A. & Gmeinwieser, J. Three-dimensional MP-RAGE – an alternative to conventional three-dimensional FLASH sequences for the diagnosis of viscerocranial tumours? Br. J. Radiol. 68, 1316-1324 (1995).

2. Deichmann, R., Schwarzbauer, C., Turner, R. Optimisation of the 3D MDEFT sequence for anatomical brain imaging: technical implications at 1.5 and 3 T. NeuroImage 21, 757-767 (2004).

3. Jack, C.R. Jr. et al. The Alzheimer's Disease Neuroimaging Initiative (ADNI): MRI methods. J. Magn. Reson. Imaging 27, 685-691 (2008).

4. van der Kouwe, A.J., Benner, T., Salat, D.H. & Fischl, B. Brain morphometry with multiecho MPRAGE. NeuroImage 40, 559-569 (2008).

5. Berkovic, S.F. et al. Hippocampal sclerosis in temporal lobe epilepsy demonstrated by magnetic resonance imaging. Ann. Neurol. 29, 175-182 (1991).

6. Coan, A.C., Kubota, B., Bergo, F.P., Campos, B.M. & Cendes, F. 3T MRI quantification of hippocampal volume and signal in mesial temporal lobe epilepsy improves detection of hippocampal sclerosis. Am. J. Neuroradiol. 35, 77-83 (2014).

7. Smith, S.M. et al. Advances in functional and structural MR image analysis and implementation as FSL. NeuroImage 23, S208-S219 (Suppl. 1, 2004).

8. Smith, S.M. Fast robust automated brain extraction. Human Brain Mapp. 17, 143-155 (2002).

9. Zhang, Y., Brady, M. & Smith, S. Segmentation of brain MR images through a hidden Markov random field model and the expectation-maximization algorithm. IEEE Trans. Med. Imag. 20, 45-57 (2001).

10. Fischl, B. et al. Whole brain segmentation: automated labeling of neuroanatomical structures in the human brain. Neuron 33, 341-355 (2002).

11. Fischl, B. et al. Sequence-independent segmentation of magnetic resonance images. NeuroImage 23, S69-S84 (Suppl 1, 2004).

12. Pardoe, H.R., Pell, G.S., Abbott, D.F., &Jackson, G.D. Hippocampal volume assessment in temporal lobe epilepsy: How good is automated segmentation? Epilepsia 50, 2586-2592 (2009).

13. Kim, H. et al. Automatic hippocampal segmentation in temporal lobe epilepsy: impact of developmental abnormalities. NeuroImage 59, 3178-3186 (2012).

14. Rummel, C., McKinley, R., Wagner, F., Salmen, A., Chan, A., Wiest, R. A fully automatic pipeline for surface-based morphometry in individual patients. under review (2017).

15. Dale, A.M., Fischl, B. & Sereno, M.I. Cortical surface-based analysis. I. Segmentation and surface reconstruction. NeuroImage 9, 179 (1999).

16. Fischl, B., Sereno, M.I. & Dale, A.M. Cortical surface-based analysis. II: Inflation, flattening, and a surface-based coordinate system. NeuroImage 9, 195 (1999).

17. Fischl, B., Sereno, M.I., Tootell, R.B. & Dale, A.M. High-resolution intersubject averaging and a coordinate system for the cortical surface. Hum. Brain Mapp. 8, 272 (1999).

18. Fischl, B. & Dale, A.M. Measuring the thickness of the human cerebral cortex from magnetic resonance images. Proc. Natl. Acad. Sci. U.S.A. 97, 11050 (2000).

19. Pienaar, R., Fischl, B., Caviness, V., Makris, N. & Grant, P.E. A methodology for analyzing curvature in the developing brain from preterm to adult. Int. J. Imaging Syst. Technol. 18, 42-68 (2008).

20. Salat, D.H. et al. Age-associated alterations in cortical gray and white matter signal intensity and gray to white matter contrast. NeuroImage 48, 21-28 (2009).

21. Grimes, D.A., Schulz, K.F. Uses and abuses of screening tests. Lancet 359, 881-884 (2002).

22. Watson, E. et al. The accuracy and efficacy of screening tests for Chlamydia trachomatis : a systematic review. J. Med. Microbiol. 51, 1021–1031 (2002).

23. Maxim, L.D., Niebo, R. Utell, M.J. Screening tests: a review with examples. Inhal Toxicol. 26, 811–828 (2014).

24. Donovan, L. et al. Screening Tests for Gestational Diabetes: A Systematic Review for the U.S. Preventive Services Task Force. Ann. Intern. Med. 159 (2013).

25. DeSisto, C.L., Kim, S.Y., Sharma, A.J. Prevalence Estimates of Gestational Diabetes Mellitus in the United States, Pregnancy Risk Assessment Monitoring System (PRAMS), 2007–2010. Prev. Chronic. Dis. 11, 130415 (2014).
